# Modifiable dementia risk factors associated with objective and subjective cognition

**DOI:** 10.1002/alz.13885

**Published:** 2024-10-09

**Authors:** Anna Marie Rosická, Vanessa Teckentrup, Sol Fittipaldi, Agustin Ibanez, Andrew Pringle, Eoghan Gallagher, Anna Kathleen Hanlon, Nathalie Claus, Cathal McCrory, Brian Lawlor, Lorina Naci, Claire M. Gillan

**Affiliations:** ^1^ School of Psychology, Trinity College Dublin Dublin Ireland; ^2^ Global Brain Health Institute, Trinity College Dublin Dublin Ireland; ^3^ Latin American Brain Health Institute (BrainLat) Santiago Chile; ^4^ Department of Psychology Chair of Clinical Psychology & Psychological Treatment LMU Munich Munich Germany; ^5^ Department of Medical Gerontology The Irish Longitudinal Study on Ageing School of Medicine, Trinity College Dublin Dublin Ireland

**Keywords:** age interactions, cognitive flexibility, cognitive impairment, cross‐sectional study, depression, executive function, model‐based planning, modifiable risk factors for dementia, smartphone assessment, smartphone data collection, subjective cognitive complaints, subjective memory problems, visual working memory

## Abstract

**INTRODUCTION:**

Early detection of both objective and subjective cognitive impairment is important. Subjective complaints in healthy individuals can precede objective deficits. However, the differential associations of objective and subjective cognition with modifiable dementia risk factors are unclear.

**METHODS:**

We gathered a large cross‐sectional sample (*N* = 3327, age 18 to 84) via a smartphone app and quantified the associations of 13 risk factors with subjective memory problems and three objective measures of executive function (visual working memory, cognitive flexibility, model‐based planning).

**RESULTS:**

Depression, socioeconomic status, hearing handicap, loneliness, education, smoking, tinnitus, little exercise, small social network, stroke, diabetes, and hypertension were all associated with impairments in at least one cognitive measure. Subjective memory had the strongest link to most factors; these associations persisted after controlling for depression. Age mostly did not moderate these associations.

**DISCUSSION:**

Subjective cognition was more sensitive to self‐report risk factors than objective cognition. Smartphones could facilitate detecting the earliest cognitive impairments.

**Highlights:**

- Smartphone assessments of cognition were sensitive to dementia risk factors.
- Subjective cognition had stronger links to most factors than did objective cognition.
- These associations were not fully explained by depression.
- These associations were largely consistent across the lifespan.

## 1 BACKGROUND

Cognitive impairments are linked to important brain health and wellbeing outcomes, and thus their early detection and prevention have become a major public health concern. One key area of focus has been on the role of potentially modifiable social, health, and lifestyle factors in helping to maintain cognitive abilities as our populations age.^1^, ^2^ Cognitive impairments can be objectively measured through standardized cognitive tests, but also subjectively perceived, typically in the form of subjective memory complaints. Understanding how to preserve both objective and subjective cognition is important, as each has been shown to have a major impact on wellbeing,^3^ daily functioning,^4^ and serious brain health outcomes like dementia.^5^ For example, a decline in performance on objective tests of memory and executive function can precede dementia onset by up to a decade.^6^ Likewise, even before cognitive impairments can be objectively measured, subjective perceptions can reflect the earliest stages of cognitive decline,^5^ and objectively unimpaired older adults who have subjective cognitive complaints are twice as likely to develop dementia than those who do not.^7^ However, neither is fully deterministic – not everyone with subjective cognitive complaints will eventually develop an objective form of cognitive decline,^7^ and more advanced objective cognitive impairment is typically accompanied by a lack of subjective insight into one's cognitive capacity, rendering subjective assessments less reliable.^8^ Thus, although objective and subjective cognitive assessments overlap considerably in their functional significance, they have a surprisingly low correlation on the individual level,^9^ which can additionally depend on age, gender, and culture.^10^ This low correlation may be because each reflects different stages of cognitive decline; subjective cognitive complaints could represent the earliest stages of cognitive decline that anticipate more consolidated, objective deficits.^5^, ^8^ By the time objective deficits emerge, a deterioration in metacognitive processes or other comorbidities like depression might affect the accuracy of subjective assessments.^11^, ^12^ Another possibility is that cross‐sectional objective assessments cannot distinguish low “premorbid” ability from cognitive decline,^5^ and so subjective cognitive complaints may be more sensitive because they reflect within‐person cognitive changes that would go undetected by objective assessments administered at one moment in time.^13^

With the importance of these different facets of cognition in mind, research has established robust associations between both objective and subjective cognition and a variety of potentially modifiable risk factors for dementia. Diabetes,^14^ history of stroke,^15^ hearing loss,^16^ tinnitus,^17^ depression,^18^ social engagement,^19^ hypertension,^20^ physical inactivity,^21^ smoking,^22^ socioeconomic status (SES),^23^ and educational attainment^24^ have all been linked to differences in performance on objective tests of executive function. Similarly, subjective cognitive complaints have also been linked to diabetes,^25^, ^26^ history of stroke,^27^ hearing loss,^28^ tinnitus,^29^ depression,^30^ and reduced social engagement.^31^, ^32^ However, the evidence is more mixed for other risk factors like hypertension,^25^ physical inactivity,^28^, ^32^, ^33^, ^34^ smoking,^34^, ^35^ SES,^36^, ^37^ and educational attainment.^28^, ^31^, ^36^ Crucially, it is currently unclear how these risk factors differentially impact objective versus subjective cognition, if certain aspects of objective cognition (eg, working memory, cognitive flexibility, or planning) are more or less affected by dementia risk factors than subjective cognition, and how these relationships change across the lifespan.^1^, ^2^

To address this, we gathered a large multivariable, cross‐sectional dataset from thousands of so‐called citizen scientists participating in research via a smartphone app, Neureka. Smartphone‐based remote assessments such as these are becoming increasingly popular tools for research and a promising way to detect both subjective and objective cognitive impairments, as they can easily combine short screening of subjective cognitive complaints with brief, gamified objective assessments of relevant aspects of cognition.^10^, ^38^, ^39^ A growing body of research shows good validity and acceptability of smartphone assessments in the field of cognitive decline (see Whelan et al^40^ for a review). The benefits of smartphone‐based assessment include low cost, low burden for examiners and examinees, scalability, accessibility, and ease of repeated testing.^41^ Here, we leveraged these features to gather rich data from a large, dementia‐free sample spanning the entire adult age range. From each participant we acquired information about previously established dementia risk factors, gamified objective assessments of cognition, and subjective cognitive complaints. Given the importance of objective executive function in daily functioning^4^ and early detection of dementia,^6^ we focused on multiple aspects of executive function, namely, working memory, cognitive flexibility, and model‐based planning. Secondary analyses also tested whether the associations between risk factors and cognition might be explained by individual differences in depression symptom severity,^26^ one of the best‐established correlates of subjective complaints.^30^

## 2 METHODS

### 2.1 Participants

Between June 2020 and July 2023, *N* = 9918 members of the public engaged with a module called “Risk Factors” in the smartphone app Neureka, which contains a battery of self‐report questionnaires and gamified cognitive assessments. Among these participants, 66.31% had missing data in at least one part of the battery. In the current study, we only used data from the first fully completed “Risk Factors” attempt linked to each participant's app account, resulting in a total sample of *N* = 3341 full completers. The full completers did not significantly differ from the remaining participants in either age (*M*_complete _= 45.69, *M*_incomplete_ = 45.62, *t *[6845] = 0.23, *p* = 0.819) or the prevalence of self‐reported memory problems (*X* ^2^ [1, *N* = 9918] = 0.01, *p* = 0.998). We further excluded participants with a self‐reported diagnosis of dementia (*n* = 6) or those who preferred not to state their gender (*n* = 8) as the sample size for those groups was too small to draw any meaningful conclusions, resulting in the final *N* = 3327 participants. The demographic characteristics of the final sample are summarized in Table 1. A histogram showing the age distribution of the sample is available in Supplement S1. Participants came from 54 different countries, whereby the most frequent countries were the United Kingdom (*n* = 1650), the United States (*n* = 936), Ireland (*n* = 443), Canada (*n* = 57), and Germany (*n* = 46). Of the final sample, 2986 (89.8%) participants reported having English as their first language (see Supplement S2 for sensitivity analyses with this reduced sample of native speakers). The Neureka project was approved by the research ethics committee of the School of Psychology, Trinity College Dublin (approval number: SPREC072019‐01). Data collected through Neureka is stored and processed in line with the EU General Data Protection Regulations.

**RESEARCH IN CONTEXT**

1. **Systematic review**: The authors reviewed existing literature using primarily Google Scholar. Although many potentially modifiable dementia risk factors have been identified, there is a lack of comprehensive studies comparing their associations with different aspects of objective versus subjective cognition in healthy adults.
2. **Interpretation**: The findings show that smartphone‐based cognitive assessments, including gamified objective tests of executive functions and single‐item subjective assessment of memory, are sensitive to a range of established dementia risk factors. The pattern of associations was largely consistent across the lifespan, meaning they did not match the sensitive windows proposed by the life‐course model of risk factors for dementia. Overall, subjective memory problems were more sensitive to most risk factors compared to objective tests.
3. **Future directions**: Future studies should use longitudinal and quasi‐experimental designs to test for reverse causation and to further inform personalized intervention strategies. Extending focus to objectively assessed risk factors would help explain the differences presented here.

**TABLE 1:** Demographic characteristics of study participants (*N* = 3327).

Variable	Details	Descriptive statistics
**Age**	M (± SD)	45.74 (± 14.55)
	Range	18 to 84
**Gender**	Cisgender female	2160 (64.9%)
	Cisgender male	1099 (33.0%)
	Non‐cisgender ^a^	68 (2.0%)
**Education**	No formal education	135 (4.1%)
	Lower secondary	639 (19.2%)
	Upper secondary	1376 (41.4%)
	University/college degree or equivalent	894 (26.9%)
	Master's degree	243 (7.3%)
	PhD	40 (1.2%)

^a^ The “non‐cisgender” category includes participants who stated their gender is “transgender male,” “transgender female,” or “non‐binary.” Participants who preferred not to state their gender (ie, 0.4% of all sign‐ups) were not included in the current study.

### 2.2 Procedure

The Neureka app was developed in‐house by our research team at Trinity College Dublin, Ireland, with the aim of facilitating large‐scale online research on brain health. Prior to its public release, the app had been focus‐grouped, piloted, validated, and iteratively improved in smaller paid research studies, and its validation continues as new modules are added to the app on an ongoing basis. The app is currently free to download from Google Play Store (Android users) and App Store (iOS users). Participants are recruited through a variety of methods, including radio and television publicity, ads on the Google Play Store, and organized efforts such as the SciStarter project. Some participants are recruited as a part of smaller paid research studies at Trinity College Dublin, Ireland. All users of the Neureka app read a common information sheet and provide digital consent to participate in brain health and cognitive neuroscience research. During registration, participants select their age, gender, education, and country. Participants must be at least 18 years old to participate in research on the app. After registration, participants can complete a variety of Science Challenge modules in the app at their own leisure, without financial compensation. Data for this study come from the Risk Factors Challenge, a module in the app that focuses on assessing dementia‐related risk factors and cognition. It includes three gamified cognitive assessments that assess objective cognition (memory, cognitive flexibility, and model‐based planning; see Supplement S3A,B for more details on task validation) and two sets of questionnaires that assess subjective cognition together with a set of potentially modifiable risk factors for dementia (more detail provided in the following sections). The three gamified cognitive assessments and two sets of questionnaires are presented to participants in a semi‐randomized order, whereby a game is always delivered first, followed by a set of questionnaires, followed by a second game, followed by another set of questionnaires, and ending with a final game. The module takes approximately 45 minutes to complete in full and can be completed in one sitting or with breaks, over an extended period of time.

### 2.3 Measures

#### 2.3.1 Visual working memory

**FIGURE 1:**
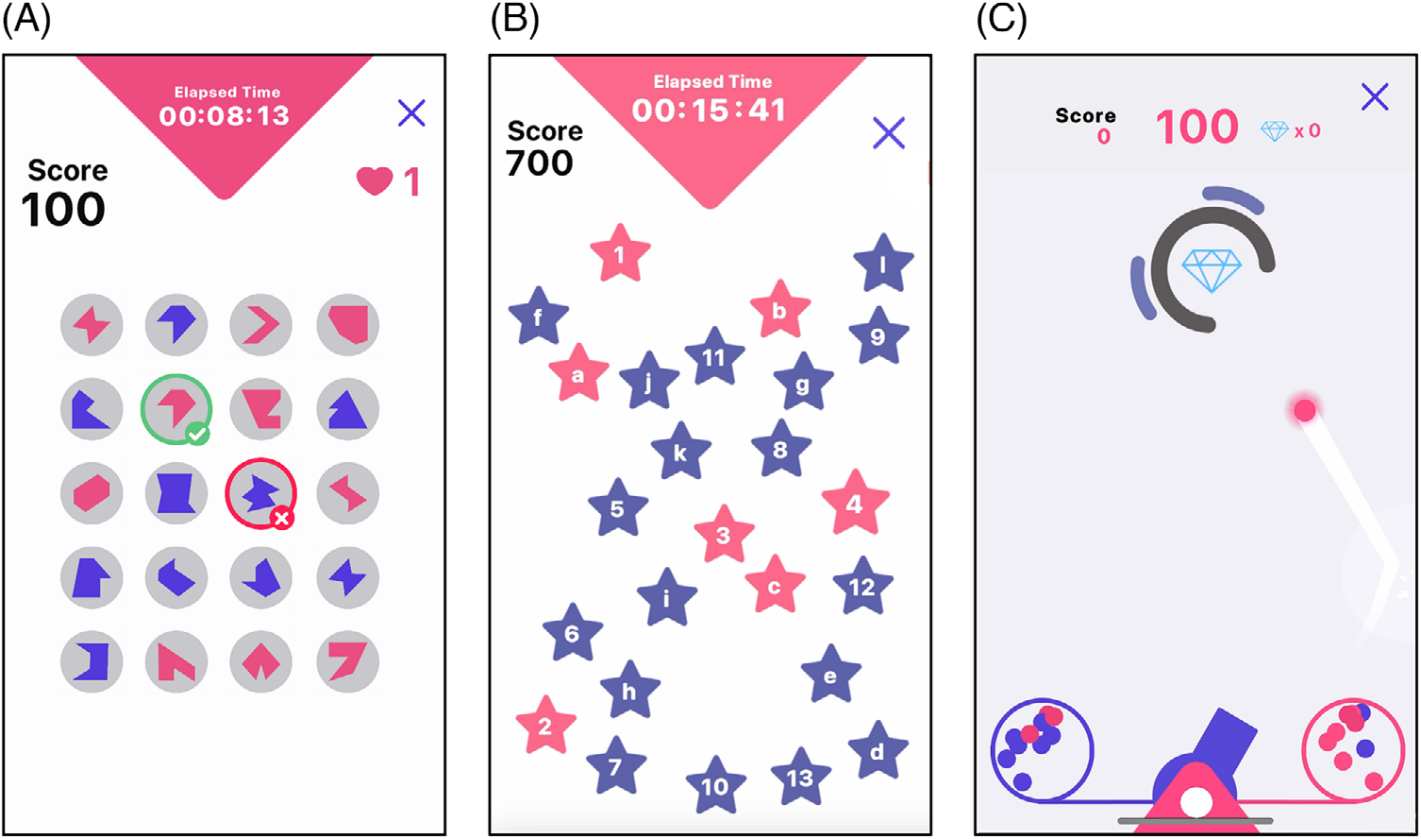
Screenshots from gamified cognitive assessments used in current study. (A) The game “Memory Match” assesses visual working memory. Participants must recognize a set of previously presented shapes or letters and selected them from a grid. (B) The game “Star Racer” assesses cognitive flexibility. Participants must tap stars in ascending order, alternating between numbers (1 to 13) and letters (a to l). (C) The game “Cannon Blast” assesses model‐based planning. Participants must pick one of two ball containers, aim a cannon, and shoot balls to collect diamonds, but their success depends on their ability to adjust to changing probabilities and picking the right container.

Visual working memory was assessed using a visual search game called Memory Match (Figure 1A), which was loosely based on the Visual Short‐Term Memory Binding Task (VSMBT).^42^ See Supplement S3A for a validation study comparing the gamified task to a traditional binding task. This game starts after a set of self‐paced instruction screens (Supplement S4A). In each trial, participants need to memorize an array of symbols and then select the previously presented study symbols from a 4 × 5 grid (Supplement S4B). Like the traditional binding task, this task design incorporates non‐binding (symbol memory only) conditions as well as binding conditions, where both the color and the symbol must be remembered. Memory Match additionally includes two stimulus types (ie, letters vs abstract shapes) and three difficulty levels with a varying study set size (ie, remembering two, three, or four symbols; see Supplement S4C for an example of the stimuli used in each trial type). These manipulations served to increase the task's sensitivity at both the low and high end of performance and to improve gameplay experience through variety. In line with gamification principles, participants in Memory Match also receive immediate visual and sound feedback on their performance, collect points throughout the task, and are given a certain number of “lives” per trial that they can lose in case of a mistake. The smallest study set size trials (ie, two symbols to remember) are presented first, and the set size increases sequentially throughout the game. As more stimuli are to be remembered in the three‐ and four‐set rounds, more lives are granted in these levels (*N* lives = *N* targets). Performance on this task is measured as the proportion of correctly memorized symbols, given the number of guesses the participant has made (ie, correct responses/correct responses + errors). Given that the search grid remains at 4 × 5 even when the study set size increases to four, random responding leads to different accuracy scores on each level. Specifically, chance‐level accuracy is 0.07, 0.12, and 0.17 for the two‐, three‐, and four‐set size levels, respectively. No symbol or color is repeated within any given study array, but they do repeat within the test displays of the binding condition, as “lures,” that is, symbols that match the target in one dimension (color or shape) but not both. The trial ends when participants select all the correct symbols or lose all their lives for the given trial, whichever comes first. There are two repeats of every trial type (2 binding conditions x 2 stimulus types x 3 study set sizes) totaling 24 trials per participant. Mean accuracy at the task was 80% (standard deviation [SD] = 8%) in the total sample, with a range of 25% to 100%.

#### 2.3.2 Cognitive flexibility

Cognitive flexibility was assessed through the game Star Racer (Figure 1B), which is a gamified and extended version of the Trail‐Making Test (TMT).^43^ See Supplement S3A for a validation study comparing it to the traditional TMT. Like the traditional TMT, Star Racer is divided into forms A and B but contains multiple “runs” of each form. The task begins with a set of self‐paced tutorial screens and practice runs of each version (Supplement S5A), which are then followed by three runs of each version (always ordered A‐B‐A‐B‐A‐B; Supplement S5B). In the A version of the game, participants are presented with a screen of 25 blue stars with numbers and are required to tap them in ascending order as quickly as possible. In the B version, the screen contains 25 stars labeled with either letters or numbers and participants must alternate between them as they ascend. Each run starts with a 3 second countdown. Participants earn points throughout the task for each correct selection (indicated by the star turning pink) and lose points for elapsed time and an incorrect selection (indicated by the star turning red, shaking, and then reverting to blue again), with their score displayed on screen throughout and summarized at the end of each run. Participants cannot progress unless they correct any erroneous selections. Performance is calculated for versions A and B separately as mean time to complete all runs of the given type, resulting in two separate outcome variables (A: processing speed and B: cognitive flexibility). However, these two variables are strongly correlated (here, *r* [3325] = 0.77, *p <* 0.001). In the current study, we focused on cognitive flexibility as a measure of executive function. We did not use a B−A difference score to ensure adequate measure reliability, which tends to be problematic for difference scores.^44^ Additionally, to identify inattentive players in this remotely administered task, we applied conservative time cut‐offs: Individual runs with completion times of 100 or more seconds (A version) or 300 or more seconds (B version) were excluded; in such cases, task performance was calculated as a mean time to complete the remaining trials. These cut‐offs were based on approximately double the median completion times for the oldest and least educated group in a normative study of the traditional task.^45^ The original mean time to complete Star Racer B was 64.31 s (SD = 24.56) with a range of 19.27 to 381.87 s, and following application of the cut‐offs, this changed to the final 63.28 s (SD = 21.73) with a range of 19.27 to 204.37 s.

#### 2.3.3 Model‐based planning

Model‐based (also known as goal‐directed) planning was assessed through a game called Cannon Blast (Figure 1C), which is a gamified version of the Two‐Step Reinforcement Learning Task with a recent extensive validation.^39^, ^46^ See Supplement S3B for a detailed description of the outcome measure and the task. In brief, the game requires participants to aim and shoot balls from a cannon to hit diamonds, for which they earn reward points. Sometimes the balls are “bad” and explode before hitting the target and participants can use statistical features of the task to avoid receiving these balls. Specifically, they can keep a basic model‐free policy that encourages them to repeat actions that lead to good balls and switch from actions that lead to bad ones. Alternatively, they can incorporate higher‐order knowledge of the task structure to refine this as a model‐based policy. The outcome measure of Cannon Blast is the model‐based index, which is an output of a hierarchical logistic regression analysis calculated across all 200 trials of the game available in risk factors.^39^ In this analysis, we measured how much the choice to repeat the action of a given trial is influenced by (i) whether that choice resulted in a good ball on the last trial (model‐free) and (ii) whether it is qualified by the task structure. The mean model‐based index value in the current sample was 0.27 (SD = 0.33) with a range of 0.46 to 2.22.

#### 2.3.4 Subjective memory problems

Subjective memory problems were assessed with one questionnaire item within the Risk Factors Challenge. Participants could respond “Yes” or “No” to the question “Do you currently experience memory problems?” This single‐item screener was the same as that used in the PREVENT study.^47^ In the current sample, 1,348 (40.5%) individuals reported subjective memory complaints.

#### 2.3.5 Risk factors for dementia

A set of social, health, and lifestyle factors, based on the risk factors for brain health identified previously,^1^, ^17^, ^48^, ^49^, ^50^ was assessed via questionnaires in the “Risk Factors” challenge. Specifically, we measured educational attainment, SES, depression, loneliness, social network size, hearing handicap, tinnitus, stroke, diabetes, hypertension, smoking, exercise, and family history of dementia. Details on the risk factor measures are summarized in Table 2.

**TABLE 2:** Description of risk factor measures.

Measure	Description/question(s)	Descriptive statistics^a^	Reference
Education	0 = No formal education 1 = Lower secondary education 2 = Upper secondary education 3 = University/College degree or equivalent 4 = Master's degree or equivalent 5 = PhD or equivalent	M = 2.82 SD = 1.00 Range = 0 to 5	N/A
Socioeconomic status (SES)	The Subjective SES scale: Participants were asked to place themselves on a ladder representing where people stand (in the country they live in) with respect to money, education, and respected jobs	M = 5.95 SD = 1.77 Range = 1 to 10	^51^
Depression	Center for Epidemiologic Studies Depression (CES‐D) scale: a 20‐item self‐report scale for measuring depressive symptoms in the general population, which asks about the frequency of symptoms over the past week	M = 17.96 SD = 13.59 Range = 0 to 60	^52^
Loneliness	UCLA Loneliness Scale: a 20‐item self‐report scale for measuring general loneliness	M = 21.10 SD = 15.54 Range = 0 to 60	^53^
Social network	Lubben Social Network Scale: a 6‐item self‐report measure of social engagement with family and friends	M = 15.96 SD = 6.46 Range = 0 to 30	^54^
Hearing handicap	Hearing Handicap Inventory for the Elderly–Screening version (HHIE‐S): a 10‐item self‐report scale measuring emotional and social consequences of hearing impairment in adults	M = 5.60 SD = 7.47 Range = 0 to 40	^55^
Tinnitus	“In the past year have you had buzzing, ringing, or noise (tinnitus) in your ears that lasts longer than 5 minutes?” (No/Yes, in the past week/Yes, not in the past week/Do not know)	Yes, in the past week/ Yes, not in the past week: 992 (29.8%) No/do not know: 2335 (70.2%)	^56^
History of stroke	“Have you ever had a stroke?” (Yes/No)	Yes: 45 (1.4%) No: 3282 (98.6%)	N/A
Diabetes	“Have you been diagnosed with diabetes by a doctor?” (Yes/No)	Yes: 174 (5.2%) No: 3153 (94.8%)	N/A
Hypertension	“Have you been diagnosed with high blood pressure (hypertension) by a doctor?” (Yes/No)	Yes: 511 (15.4%) No: 2816 (84.6%)	N/A
Smoking history	“Please indicate which of the following best describes you:” (1. Non‐smoker/ 2. Ex‐smoker/ 3. Current smoker/ 4. Unknown)	Current smoker/Ex‐smoker: 1134 (34.1%) Non‐smoker/Unknown: 2193 (65.9%)	Based on PREVENT study^47^
Exercise	Godin‐Shephard Leisure‐Time Physical Activity Questionnaire: a self‐report measure of physical activity that asks about the frequency of strenuous, moderate, and mild exercise per week	M = 32.70 SD = 25.48 Range = 0 to 119	^57^
Family history of dementia	“Was your biological mother ever diagnosed with dementia?” (Yes/No) “Was your biological father ever diagnosed with dementia?” (Yes/No)	Yes (either parent or both): 560 (16.8%) No: 271 (83.2%)	Based on PREVENT study^47^

^a^ By design, there were no missing data for either measure in the total sample.

### 2.4 Data analysis

#### 2.4.1 Data preparation

All analyses were conducted using R Statistical Software version 4.3.1.^58^ Code to reproduce the findings and figures is available at https://osf.io/vq6fa/, alongside more details on how to request data. Prior to analyses, variables were reverse‐coded when necessary, so that in all cases higher values indicate worse cognition or higher risk. Furthermore, all continuous variables (both independent and dependent) were scaled by mean‐centering and dividing by their SD within each sample. To ensure the estimates for continuous and binary categorical predictors in our models are directly comparable to each other (see Gelman^59^ for a discussion of the problem), we scaled binary variables (history of hypertension, smoking, stroke, diabetes, tinnitus, family history of dementia, or subjective memory problems) to have a SD of 1 by coding them as 0/2. Gender was contrast‐coded with “cisgender male” as the reference category. We assessed the effects of age and gender on each objective cognitive measure using a series of linear regressions and on subjective memory problems using logistic regression. For age, we included both a linear and a quadratic term (ie, age squared) to capture potential non‐linear effects of chronological age across the wide age span in our sample. Effect sizes were reported as standardized betas and odds ratios (ORs) with associated confidence intervals (CIs). Associations between cognitive outcomes (ie, visual working memory, cognitive flexibility, model‐based planning, and subjective memory problems) were assessed using point‐biserial correlation with a two‐step estimate for pairs of numeric and ordinal variables and Pearson product‐moment correlation for continuous variables.

#### 2.4.2 Primary analysis

To test the differential cognitive correlates of previously established risk factors for dementia, we ran a series of linear, respective logistic regressions, predicting objective, respective subjective cognition from each risk factor individually, controlling for gender and age. We calculated standardized betas and respective ORs with appropriate CIs for each association. Because of their different interpretation (increase in *y* per SD change in *x* vs increase in log odds of *y* per SD change in *x*), standardized betas in linear and logistic regression are not directly comparable. Therefore, to be able also to directly compare the magnitude of the risk factor effects on the continuous and categorical dependent variables, we additionally transformed the continuous dependent variables to dichotomous, resulting in new categorical measures of worse versus better cognitive flexibility, visual working memory, and model‐based planning. To ensure the group sizes (worse vs better cognition) were comparable between all cognitive measures, we used the ratio of subjective memory problems as a quantile cut‐off. That is, subjective memory problems were present in 40.5% of the sample, and so our binarization of objective cognitive scores returned 40.5% classified as having poor objective performance on visual working memory, cognitive flexibility, and model‐based planning. We then ran an additional series of logistic regressions predicting each binarized objective cognitive outcome from each risk factor individually, controlling for gender and age, and calculated ORs for each association. For each cognitive outcome, Bonferroni correction was applied to correct for multiple comparisons, dividing α = 0.05 by the number of risk factors (ie, each independent variable was understood as one comparison).

#### 2.4.3 Secondary analyses

First, we tested whether the associations between risk factors and objective and subjective cognition change after controlling for depression. We ran the same series of analyses as described in the primary analysis section, this time additionally controlling for depression as measured by the CES‐D scale. This was done for 12 of the 13 original risk factors, excluding depression itself.

Next, we tested whether the associations between all 13 risk factors and cognition differed by age. This subgroup analysis was only tested on those combinations of independent and dependent variables whose associations were previously significant. We ran the same series of regressions as in the primary analysis, predicting objective respectively subjective cognition from each of this reduced set of risk factors, controlling for gender and age, this time with an added interaction term (ie, age *x* risk factor). Bonferroni correction was applied to correct for multiple comparisons, dividing α = 0.05 by the number of interaction terms of interest (ie, each combination of independent and dependent variables was understood as one comparison).

## 3 RESULTS

### 3.1 Descriptive and demographic analyses

As has been consistently reported in the literature, objective and subjective cognition were only weakly correlated (*r *= 0.06 to 0.16; see Table 3 for correlations between all cognitive measures). The correlation matrix of all self‐reported risk factors is presented in Supplement S6.

**TABLE 3:** Correlations between cognitive measures.

	Subjective memory problems	Model‐based planning	Cognitive flexibility
Visual working memory	0.09	0.26	0.45
Cognitive flexibility	0.16	0.15	
Model‐based planning	0.06		

*Note*: Numbers represent Pearson product‐moment correlation coefficients for pairs of continuous variables and polyserial correlations for pairs of continuous and categorical variables. All coefficients are significant at *p* < .05 or lower.

**FIGURE 2:**
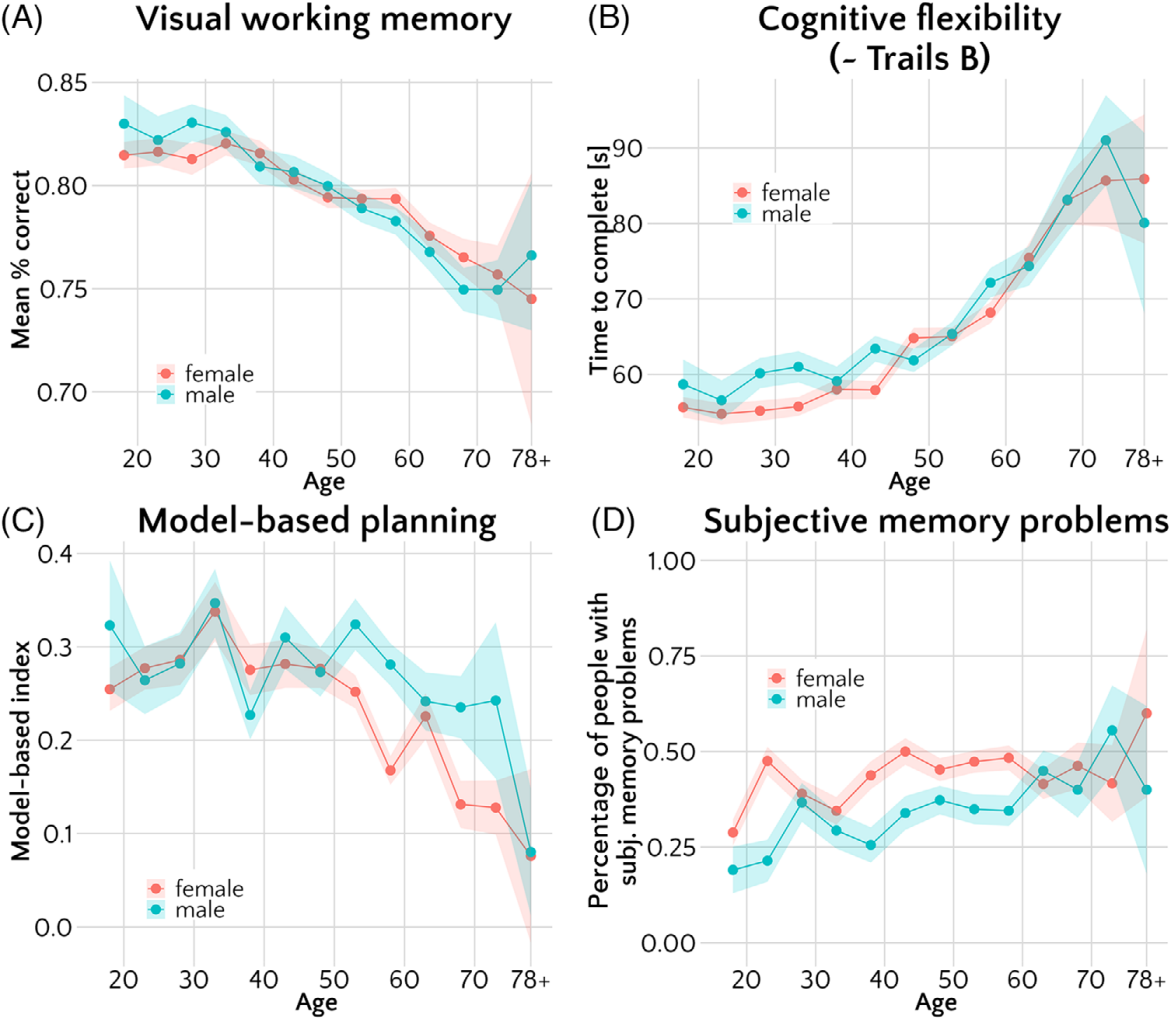
Associations of cognitive measures with age and gender. Points correspond to mean raw scores on (A) visual working memory, (B) cognitive flexibility, (C) model‐based planning, and (D) mean proportion of participants with memory problems. The means were calculated for 5‐year bins, split by gender. Error bars represent standard errors (A–C) or standard errors of proportion (D). Only cisgender participants are displayed in this plot (*N* = 3259); for plots including non‐cisgender participants please see Supplement S7.

The older the participants, the worse their objective cognitive performance across domains (standardized β [95% CI] = 0.20 [0.16, 0.23] for visual working memory, β [95% CI] = 0.34 [0.30, 0.37] for cognitive flexibility, and β [95% CI] = 0.09 [0.05, 0.12] for model‐based planning, all *p <* 0.001; see Figure 2A–C). We also found a significant quadratic effect of age on all three objective cognitive measures: The older the participants, the stronger the association between age and visual working memory (β [95% CI] = 0.07 [0.04, 0.11], *p <* 0.001), cognitive flexibility (β [95% CI] = 0.13 [0.10, 0.17], *p <* 0.001), and model‐based planning (β [95% CI] = 0.07 [0.03, 0.10], *p <* 0.001), indicating a steeper curve in older age groups. This quadratic effect was significant, even though the raw cognitive scores plateaued in the oldest age groups (ie, around 70 to 75 years), as shown in Figure 2A–C, which could suggest evidence for selection bias in the oldest participants. Of note, very few participants reported being 75 and older (*n* = 30, ie, 0.9% of the sample; see Supplement S1 for illustration). Subjective memory problems were significantly more likely in older participants (OR [95% CI] = 1.04 [1.01, 1.07], *p = *0.021; see Figure 2D). However, unlike in the objective measures, there was no significant quadratic effect of age on subjective memory problems (OR [95% CI] = 1.00 [1.00, 1.00], *p = *.122). To ensure similarity between models of objective and subjective cognition, the main covariate‐adjusted analyses (see next section) included age as a simple linear term.

As for gender, we focused only on cisgender men and women for our main analyses due to sample size. We found that cisgender women did not significantly differ from cisgender men on visual working memory (β [95% CI] = 0.00 [−0.03, 0.04]; *p = *.915; Figure 2A), but they had significantly worse model‐based planning (β [95% CI] = 0.05 [0.01, 0.08]; *p = *0.010; Figure 2C) and were more likely to report subjective memory problems (OR [95% CI] = 1.24 [1.15, 1.34], *p <* 0.001; Figure 2D). Cisgender women showed higher cognitive flexibility than men, indexed by faster game completion times (β [95% CI] = −0.04 [−0.08, −0.01]; *p = *0.012; Figure 2B). Comparisons of the cognition of transgender and non‐binary participants are difficult to make due to the very small sample size of these groups (*n* = 68, ie, 2% of the sample), but they are described in Supplement S7.

### 3.2 Risk factors and cognition

**FIGURE 3:**
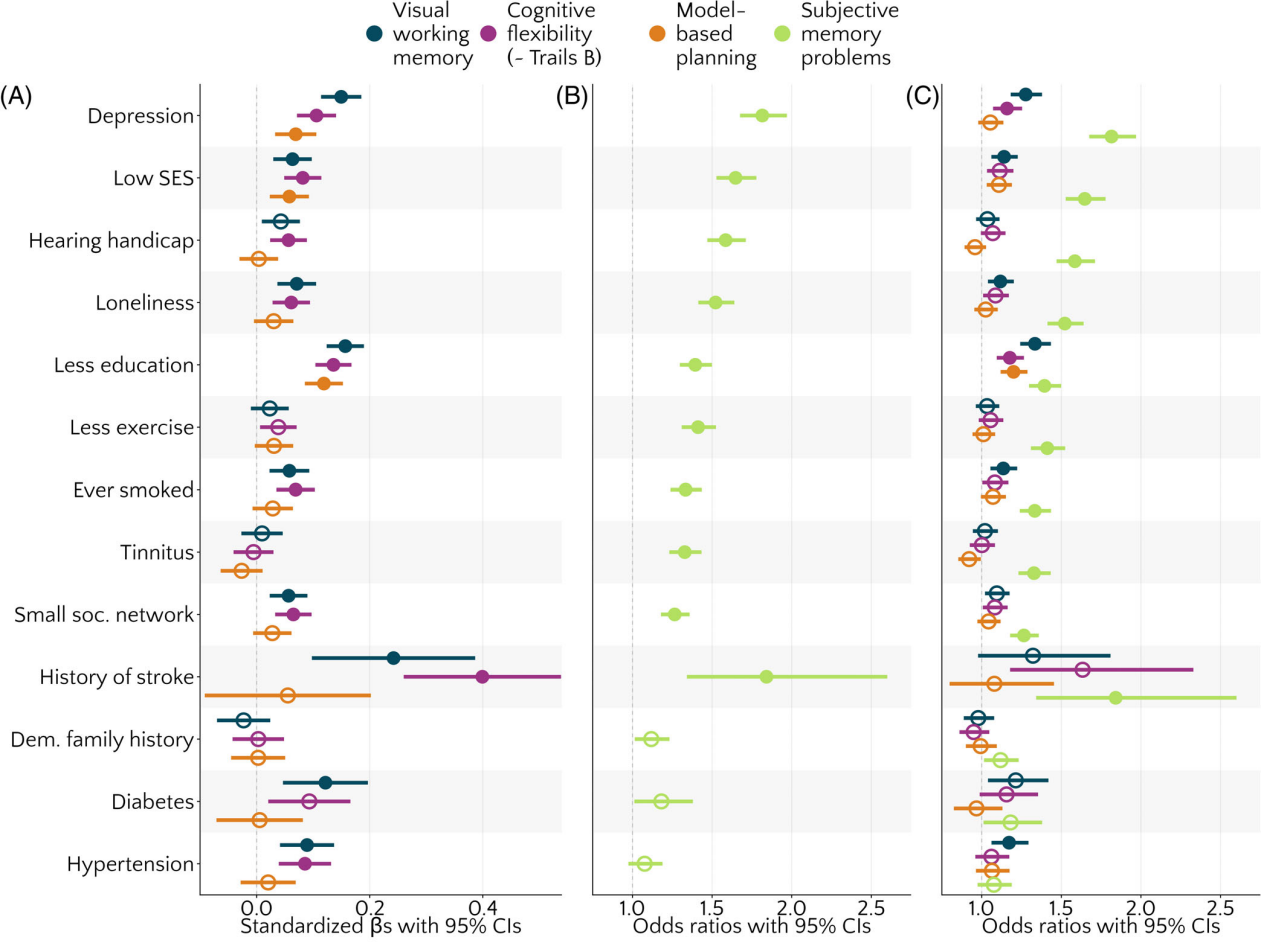
Associations between cognitive measures and risk factors, controlling for age and gender. Filled circles represent values significant at *p *< .0038, that is, after applying Bonferroni correction per dependent variable. Higher estimates indicate worse cognitive outcomes. Circles and lines represent (A) standardized beta estimates with 95% confidence intervals (CIs) or (B and C) odds ratios with 95% CIs. In (C), objective cognitive scores were binarized to enable direct comparison of odds ratios with subjective memory problems.

After applying Bonferroni correction per each dependent measure, we set our alpha level to α = 0.05/13 = 0.0038. The associations between risk factors and visual working memory, cognitive flexibility, model‐based planning, and subjective memory problems, controlling for age and gender, are visualized in Figure 3, and detailed results are presented in Table 4.

**TABLE 4 alz13885-tbl-0004:** Associations of risk factors with cognitive outcomes (*n* = 3327), controlling for gender and age or gender, age, and depression, expressed as estimates from logistic regressions (subj. memory problems) or linear regressions (all other DVs).

Risk factor	Covariates	Visual working memory			Cognitive flexibility			Model‐based planning			Subjective memory problems			
		β (SE)	*t*	*P*	β (SE)	*t*	*P*	β (SE)	*t*	*P*	β (SE)	*z*	*P*	OR [CI_95%_]
Depression	Age + gender	**0.15 (0.02)**	**8.23**	**<0.001**	**0.11 (0.02)**	**6.01**	**<0.001**	**0.07 (0.02)**	**3.72**	**<0.001**	**0.6 (0.04)**	**14.45**	**<0.001**	**1.82 [1.68, 1.97]**
Low SES	Age + gender	**0.06 (0.02)**	**3.66**	**<0.001**	**0.08 (0.02)**	**4.87**	**<0.001**	**0.06 (0.02)**	**3.28**	**0.001**	**0.5 (0.04)**	**12.89**	**<0.001**	**1.65 [1.53, 1.78]**
	Age + gender + depression	0.01 (0.02)	0.28	0.778	0.05 (0.02)	2.62	0.009	0.04 (0.02)	1.91	0.056	**0.33 (0.04)**	**7.86**	**<0.001**	**1.39 [1.28, 1.51]**
Hearing handicap	Age + gender	0.04 (0.02)	2.50	0.012	**0.06 (0.02)**	**3.40**	**0.001**	0 (0.02)	0.22	0.823	**0.46 (0.04)**	**11.89**	**<0.001**	**1.59 [1.47, 1.71]**
	Age + gender + depression	0.01 (0.02)	0.30	0.767	0.03 (0.02)	1.85	0.064	−0.01 (0.02)	−0.82	0.413	**0.35 (0.04)**	**8.79**	**<0.001**	**1.43 [1.32, 1.54]**
Loneliness	Age + gender	**0.07 (0.02)**	**4.06**	**<0.001**	**0.06 (0.02)**	**3.63**	**<0.001**	0.03 (0.02)	1.70	0.089	**0.42 (0.04)**	**11.08**	**<0.001**	**1.52 [1.41, 1.64]**
	Age + gender + depression	−0.04 (0.02)	−1.83	0.068	−0.01 (0.02)	−0.46	0.648	−0.02 (0.02)	−1.02	0.307	0.09 (0.05)	1.87	0.061	1.1 [1, 1.21]
Less education	Age + gender	**0.16 (0.02)**	**9.33**	**<0.001**	**0.14 (0.02)**	**8.34**	**<0.001**	**0.12 (0.02)**	**6.93**	**<0.001**	**0.33 (0.04)**	**9.01**	**<0.001**	**1.39 [1.3, 1.5]**
	Age + gender + depression	**0.14 (0.02)**	**8.03**	**<0.001**	**0.12 (0.02)**	**7.40**	**<0.001**	**0.11 (0.02)**	**6.37**	**<0.001**	**0.26 (0.04)**	**6.77**	**<0.001**	**1.3 [1.2, 1.4]**
Less exercise	Age + gender	0.02 (0.02)	1.37	0.172	0.04 (0.02)	2.33	0.020	0.03 (0.02)	1.77	0.077	**0.34 (0.04)**	**8.88**	**<0.001**	**1.41 [1.31, 1.52]**
	Age + gender + depression	0 (0.02)	−0.07	0.941	0.02 (0.02)	1.30	0.193	0.02 (0.02)	1.14	0.256	**0.26 (0.04)**	**6.63**	**<0.001**	**1.3 [1.2, 1.41]**
Ever smoked	Age + gender	**0.06 (0.02)**	**3.23**	**0.001**	**0.07 (0.02)**	**3.98**	**<0.001**	0.03 (0.02)	1.57	0.118	**0.29 (0.04)**	**7.66**	**<0.001**	**1.33 [1.24, 1.44]**
	Age + gender + depression	0.03 (0.02)	1.77	0.077	**0.05 (0.02)**	**2.94**	**0.003**	0.02 (0.02)	0.90	0.371	**0.2 (0.04)**	**5.16**	**<0.001**	**1.22 [1.13, 1.32]**
Tinnitus	Age + gender	0.01 (0.02)	0.52	0.604	−0.01 (0.02)	−0.30	0.763	−0.03 (0.02)	−1.40	0.162	**0.28 (0.04)**	**7.31**	**<0.001**	**1.33 [1.23, 1.43]**
	Age + gender + depression	−0.02 (0.02)	−0.82	0.412	−0.02 (0.02)	−1.29	0.196	−0.04 (0.02)	−2.03	0.042	**0.21 (0.04)**	**5.15**	**<0.001**	**1.23 [1.14, 1.33]**
Small social network	Age + gender	**0.06 (0.02)**	**3.32**	**0.001**	**0.07 (0.02)**	**3.96**	**<0.001**	0.03 (0.02)	1.61	0.108	**0.24 (0.04)**	**6.49**	**<0.001**	**1.27 [1.18, 1.36]**
	Age + gender + depression	0 (0.02)	0.19	0.852	0.03 (0.02)	1.79	0.073	0 (0.02)	0.19	0.851	0.03 (0.04)	0.85	0.397	1.03 [0.96, 1.12]
History of stroke	Age + gender	**0.24 (0.07)**	**3.29**	**0.001**	**0.4 (0.07)**	**5.63**	**<0.001**	0.06 (0.07)	0.74	0.461	**0.61 (0.17)**	**3.65**	**<0.001**	**1.84 [1.34, 2.6]**
	Age + gender + depression	0.19 (0.07)	2.64	0.008	**0.37 (0.07)**	**5.17**	**<0.001**	0.03 (0.08)	0.43	0.666	0.46 (0.17)	2.65	0.008	1.58 [1.14, 2.25]
Family history of dementia	Age + gender	−0.02 (0.02)	−0.95	0.340	0 (0.02)	0.13	0.897	0 (0.02)	0.11	0.909	0.11 (0.05)	2.25	0.024	1.12 [1.01, 1.23]
	Age + gender + depression	−0.02 (0.02)	−0.93	0.353	0 (0.02)	0.16	0.876	0 (0.02)	0.13	0.896	0.12 (0.05)	2.40	0.016	1.13 [1.02, 1.25]
Diabetes	Age + gender	**0.12 (0.04)**	**3.18**	**0.002**	0.09 (0.04)	2.52	0.012	0.01 (0.04)	0.14	0.889	0.17 (0.08)	2.11	0.035	1.18 [1.01, 1.38]
	Age + gender + depression	0.1 (0.04)	2.72	0.007	0.08 (0.04)	2.17	0.030	0 (0.04)	−0.08	0.934	0.1 (0.08)	1.25	0.211	1.11 [0.94, 1.3]
Hypertension	Age + gender	**0.09 (0.02)**	**3.65**	**<0.001**	**0.09 (0.02)**	**3.62**	**<0.001**	0.02 (0.02)	0.82	0.410	0.07 (0.05)	1.45	0.146	1.08 [0.97, 1.19]
	Age + gender + depression	**0.08 (0.02)**	**3.18**	**0.001**	**0.08 (0.02)**	**3.27**	**0.001**	0.01 (0.02)	0.59	0.553	0.03 (0.05)	0.53	0.594	1.03 [0.93, 1.14]

*Note*: Highlights indicate results significant after Bonferroni correction (*p* < 0.0038).

All three objective cognitive outcomes had significant associations with education, depression, and SES (ordered from largest to smallest effect size), in that worse cognition was associated with higher risk (Figure 3A). Model‐based planning had no other significant associations with any other risk factor. Both visual working memory and cognitive flexibility, however, were significantly associated with history of stroke and hypertension, followed by loneliness, smoking history, social network size, and hypertension, in that worse cognition was associated with higher risk. Additionally, visual working memory was significantly linked to diabetes and cognitive flexibility to hearing handicap. None of the objective cognitive measures assessed here had significant associations with tinnitus, exercise, or family history of dementia. Overall, the effect sizes were comparable for visual working memory and cognitive flexibility.

Subjective memory complaints were significantly associated with 10/13 risk factors (Figure 3B), namely, depression, SES, hearing handicap, loneliness, education, exercise, smoking history, tinnitus, social network size, and history of stroke (ordered from largest to smallest effect size). Subjective memory complaints were not significantly associated with family history of dementia, diabetes, or hypertension.

### 3.3 Comparison of effect magnitude for subjective versus objective cognition

Next, we compared the magnitude of the risk factor effects (expressed as ORs) on all cognitive outcomes, using a binarized version of the three objective cognitive measures as dependent variables in a series of logistic regressions. As illustrated in Figure 3C, objective and subjective cognitive measures differed in the strength of their associations with risk factors. Compared to the objective cognitive measures, subjective memory problems were more strongly associated with eight of 13 of the risk factors studied, namely, depression, SES, hearing handicap, loneliness, exercise, smoking, tinnitus, and social network. In case of higher risk on each of these eight factors, the odds of having subjective memory problems were increased by 27% to 82%, whereas the odds of having worse visual working memory or lower cognitive flexibility were increased only by 0% to 28% (Supplement S8). Objective and subjective cognition had equivalent associations with education, history of stroke, family history of dementia, hypertension, and diabetes, as indicated by overlapping CIs for the ORs expressing these effects. For example, in contrast to SES, which had about 50% higher OR for subjective versus objective cognitive measures, objective and subjective cognitive outcomes were comparably sensitive to education, a highly related construct. Of note, although the CIs of all measures were overlapping for hypertension, only the binarized objective measure of visual working memory was significantly associated to it, making it the only case where an objective measure outperformed subjective measures in sensitivity to risk factors when put on the same binarized scale. Detailed results are presented in Supplement S8.[Table alz13885-tbl-0004]

### 3.4 Depression‐adjusted analyses

**FIGURE 4:**
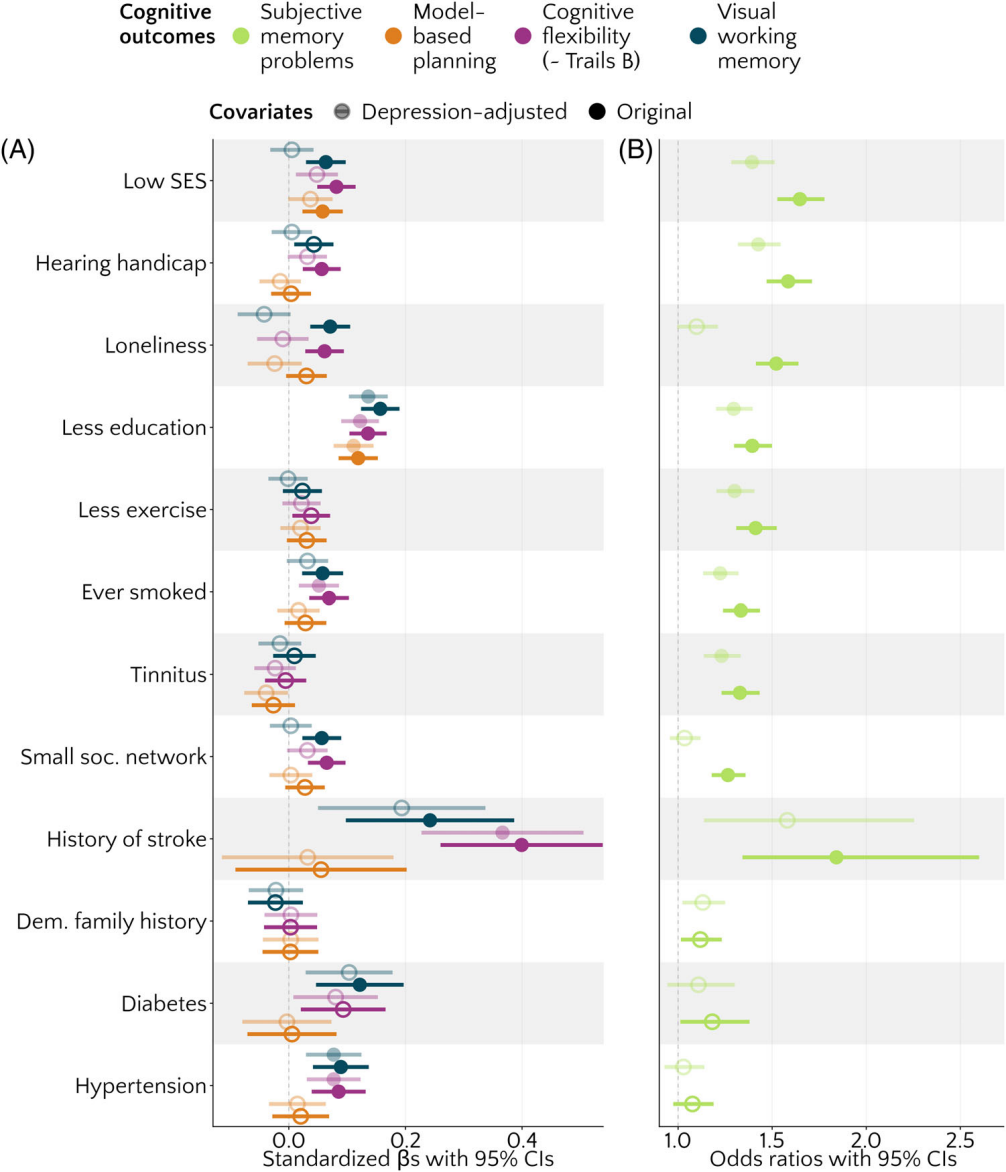
Associations between cognitive measures and risk factors, expressed as standardized beta estimates (A) or odds ratios (B) with 95% confidence intervals. Lighter shades represent depression‐adjusted analyses; darker shades represent original analyses. All analyses additionally controlled for gender and age. Filled circles represent values significant at *p < *.0038, that is, after applying Bonferroni correction per dependent variable. Higher estimates indicate worse cognitive outcomes.

To test whether depression symptoms might explain the stronger associations between subjective memory complaints and self‐report risk factors, we repeated our primary analyses controlling for depression. This was done for 12 of the 13 original analyses, excluding the analysis of depression itself. As seen in Figure 4, in general, adding depression as a covariate nominally decreased the magnitude of associations between most risk factors and subjective memory problems. However, it did not render them non‐significant, in most cases: The total number of significant associations decreased from nine out of 12 to six out of 12 risk factors. The associations with loneliness, social network, and history of stroke were no longer statistically significant at *p <* 0.0038 after adjusting for depression. The full results are presented in Table 4.

For objective cognitive outcomes, where the effects were more modest to begin with, the number of significant associations with risk factors reduced substantially when controlling for depression, here from eight out of 12 to two out of 12 for visual working memory, from eight out of 12 to four out of 12 for cognitive flexibility, and from two out of 12 to one out of 12 for model‐based planning. Of note, adjusting for depression removed the associations of all objective cognitive outcomes with SES, whereas the associations between all objective cognitive outcomes and education held. See Table 4 for detailed results.

### 3.5 Interactions between age and risk factors

In the regression models described in the primary analysis, 31 risk factors showed a significant association with cognitive outcome. For these 31 combinations of risk factors and cognitive outcomes, we ran an additional series of models with the added term of the risk factor's interaction with age, setting a new alpha level to *α* = 0.05/31 = 0.0016 to control for multiple comparisons. Broadly, we observed that almost none of these effects were moderated by age. Only one factor showed a significant interaction with age: Smoking history differed in its association with subjective memory problems by age (OR [95% CI] = 0.87 [0.81, 0.94], *p <* 0.001), showing stronger associations in younger adults than older adults (Supplement S9). No other risk factor showed a significant interaction with age (see Supplement S10 for a complete list of interaction terms of these 31 models).

## 4 DISCUSSION

Objective and subjective cognitive impairments are independent predictors of dementia, yet their differential sensitivity to previously established risk factors is little understood. Here, we estimated the pattern and magnitude of associations between potentially modifiable dementia risk factors and subjective memory problems compared to multiple aspects of objective cognition using large‐scale cross‐sectional data (*N* = 3327) gathered across the adult lifespan via smartphone. Consistent with previous findings,^9^ objective cognitive measures were only weakly correlated with subjective memory problems. Both were more frequent in older participants, but women exhibited a higher frequency of subjective, but not objective, cognitive impairments, in line with prior research.^32^ Both objective and subjective cognition were associated with depression, SES, and education, and, with the exception of the objective measure of model‐based planning, all measures were additionally associated with loneliness, social network size, history of smoking and stroke, and hypertension. Interestingly, several risk factors were selectively linked with objective or subjective cognition; only working memory or cognitive flexibility were associated with hypertension and diabetes, whereas only subjective impairments were associated with less exercise and tinnitus. Crucially, our findings suggest that across the adult lifespan, subjective memory complaints are considerably more related to self‐reported risk factors for dementia than objective differences in cognitive ability assessed via smartphone. When put on the same binarized scale, subjective memory was more strongly associated than any objective test with eight out of 13 factors, namely, depression, SES, hearing handicap, loneliness, tinnitus, exercise, and social network, and the magnitude of associations was up to 50% stronger for subjective memory. It was outperformed by an objective cognitive measure (visual working memory) only in the case of hypertension. This implies high sensitivity of subjective cognitive assessment to dementia risk factors in healthy individuals, possibly reflecting relatively higher usefulness of subjective assessments in the earliest stage of cognitive decline before impairments can be objectively detected, a finding further supported by prior evidence.^5^, ^7^

One possible explanation for the current findings is that the discrepancy between objective and subjective cognition could be driven by negative interpretive bias. That is, those who view the world and themselves more negatively might be more likely to respond negatively on subjective scales, biasing the measurement of risk factors and cognitive impairments alike. We repeated our analyses controlling for depression and found that magnitudes of effects decreased across the board when depression was included in the model. However, despite this reduction in magnitude, associations between most risk factors and subjective memory remained significant, whereas most associations with objective cognitive measures were rendered non‐significant. The two factors that changed most substantially when depression was included were loneliness and social network size, which no longer showed associations with either objective or subjective cognition after controlling for depression. This suggests that the link between these measures of social engagement and both objective and subjective brain health may be mediated by depression, consistent with prior research.^60^, ^61^, ^62^ Overall, the impact of depression on these models was marked, but not complete, suggesting that depression may be an important element of the causal path between self‐report risk factors and objective cognitive impairments, but longitudinal or quasi‐experimental studies are needed to understand how these are precisely related.

We did not observe associations between most risk factors and model‐based planning, which is to our knowledge the first time this question has been posed. We were interested in addressing this as previous research suggested that model‐based planning declined in older age^63^ but also critically relied on the healthy functioning of the hippocampus.^64^ However, contrary to our prediction, model‐based planning was only associated with education, depression, and SES. It is unlikely these null effects can be attributed to a lack of sensitivity of our smartphone task, as a recent paper showed it is sensitive to individual differences in compulsivity,^39^ replicating work with the classic task.^65^ Nonetheless, it must be acknowledged that all four cognitive assessments used in this study differed in not just their degree of objectivity but also their duration and psychometric properties, and direct comparisons are subject to those caveats. Despite this, it is striking that the largest correlates were observed for subjective memory, which was assessed using a single‐item self‐report assessment. All three cognitive tests, in contrast, were validated against traditional benchmarks, aggregated across many trials, and included several features to improve their performance over traditional versions (eg, reducing ceiling effects, increasing reaction time measurement precision).

This study raises the general question of whether objective assessments of cognition or its subjective perceptions should be prioritized as outcomes in research and public interventions. Objective measures gathered at a single time point cannot identify cognitive decline because of premorbid differences in ability; this might explain the overall reduced signal observed here for objective cognitive tests. However, it is notable that objective assessments of cognitive flexibility and working memory were nonetheless both sensitive to hypertension and only the latter to diabetes, while subjective cognition was not. This could be because cardiovascular factors and associated structural changes to brain health affect cognitive abilities more directly,^20^ while causal paths for lifestyle, sociodemographic, and social factors might be more complex. Although cross‐sectional, these findings may provide a useful starting point for thinking about such paths. For example, our findings with respect to depression support the idea that the association between low social involvement and both objective and subjective cognition could be explained by depression.^66^

The association of cognition with most risk factors in the current study did not differ by age, except for subjective memory problems and smoking. This finding contrasts with the life‐course model of risk factors for dementia,^1^, ^2^ which posits that different periods of life might provide “sensitive windows” during which various factors might come into play. Furthermore, the only interaction effect observed in the current study was opposite to those posited by the prevailing life‐course model: We found that the association between smoking and subjective memory problems was strongest in the youngest participants, whereas previous meta‐analytical work^1^, ^2^ placed smoking as a risk factor for later life. However, another recent study using large cross‐sectional data (*N *> 40,000) collected across the lifespan found that risk factor prevalence or associations with cognition did not match the prevailing life‐course model, suggesting the mapping of “sensitive windows” might be inaccurate.^67^ Longitudinal studies using dementia incidence as an outcome could elucidate whether this mismatch generalizes beyond cognition.

This study has several limitations. First, due to its cross‐sectional nature, the risk factor variables described here could be understood as “diagnostic factors”^68^ or “risk markers”^69^ rather than true risk factors. This precludes us from drawing causal inferences about the etiology of memory impairment from this dataset alone. That said, prior work using a suite of methodologies has converged on a likely causal role for many of these risk factors in brain health and dementia.^2^, ^70^, ^71^ ^,but cf.^ ^72^, ^73^ Second, the current assessment of subjective memory problems could be improved. We relied on a common single item, binary measure of subjective memory problems without any anchor, whereas previous research showed that age‐anchored comparisons and self‐comparisons (ie, comparing own memory to that of individuals of the same age respectively to one's own memory 5 years ago) differed in their associations with depression and age.^74^, ^75^ Third, the methods of recruitment (ie, online) and data collection (ie, smartphone assessment) were likely to introduce selection bias, especially in older participants as they both require digital literacy and are probably more appealing to individuals of higher SES.^76^ The presence of such selection bias could be indicated by a biologically implausible inflection point in cognitive abilities across the adult lifespan. This was reported for another smartphone study,^77^ where cognitive performance was found to markedly rise in adults over the age of 75. In the present study, very few participants were over 70, so this bias was not very apparent. We showed an average decrease in cognitive performance even in older participants up until a plateau around 70 to 75 years of age. Nevertheless, a more subtle selection bias might exist that raises important issues for the representativeness of smartphone research in older adults. However, the remote, self‐paced nature of smartphone‐based research can make it more representative in other respects, compared to in‐person lab‐based studies.^78^ Therefore, convergence across a variety of research methods is key. Fourth, we cannot exclude the possibility that domain differences contributed to the differences between subjective and objective assessment. We focused on tasks with a strong executive function component based on prior work linking executive function to healthy brain aging,^6^, ^79^ but it is possible that an objective test of episodic verbal memory could have shown even stronger effects.^6^ Episodic memory might have also been more salient for the current participants’ self‐evaluations, possibly contributing to the discrepancy. Finally, we assessed risk factors using self‐report due to the remote nature of the study. Other research has shown different associations of objectively and subjectively measured factors with brain health outcomes. For example, informant‐based subjective hearing impairment but not objectively measured hearing loss predicted incident dementia.^80^

Despite these limitations, this study shows the respective value of objective and subjective cognitive assessment and is the first, to our knowledge, to directly compare these types of assessment on associations with previously established risk factors for dementia. This study also highlights the potential of self‐administered, smartphone‐based assessments to detect early cognitive impairment and its meaningful associations with established risk factors for dementia. This may be particularly valuable for studying how risk factors impact cognition in low‐ and middle‐income countries where access to cognitive assessment is more difficult^78^ and the relative importance of risk factors for dementia differs.^81^ Future randomized controlled trials of prevention interventions should consider including brief subjective memory assessments as cognitive outcomes, alongside objective measures.

## CONFLICT OF INTEREST STATEMENT

The authors declare no conflicts of interest. Author disclosures are available in the Supporting information.

## CONSENT STATEMENT

All human subjects provided informed consent.

## Supporting information

Supporting Information

ICMJE Disclosure Forms
