# Supplementary material for: Modifiable dementia risk factors associated with objective and subjective cognition
Source: Alzheimers Dement. 2024 Oct 9;20(11):7437–52. doi: 10.1002/alz.13885 (PMC11567824; doi:10.1002/alz.13885)
Supplement: Supplementary file 1 — Supporting Information [file ALZ-20-7437-s002.pdf]

Supplement

Table of Contents

S1: Age distribution in the final sample 2
Figure S1 2
S2: Sensitivity analysis excluding non-native speakers 3
Figure S2 3
S3: Validation of gamified cognitive measures 4
S3A Memory Match and Star Racer 4
Method 4
Figure S3. 7
Results 9
Figure S4 11
Figure S5. 12
Figure S6 13
S3B Cannon Blast 14
Figure S7. 15
S4: Task structure of Memory Match 16
Figure S8. 16
S5: Task structure of Star Racer 17
Figure S9. 17
S6: Associations between risk factor measures 18
Figure S10 18
S7: Results for non-cisgender participants 19
Figure S11 19
S8: Comparison of effect magnitude for subjective vs. objective cognition 20
Table S1. 20
S9: Smoking history x age interaction 21
Figure S12 21
S10: Interactions between age and risk factors 22
Table S2. 22
References 24

S1: Age distribution in the final sample

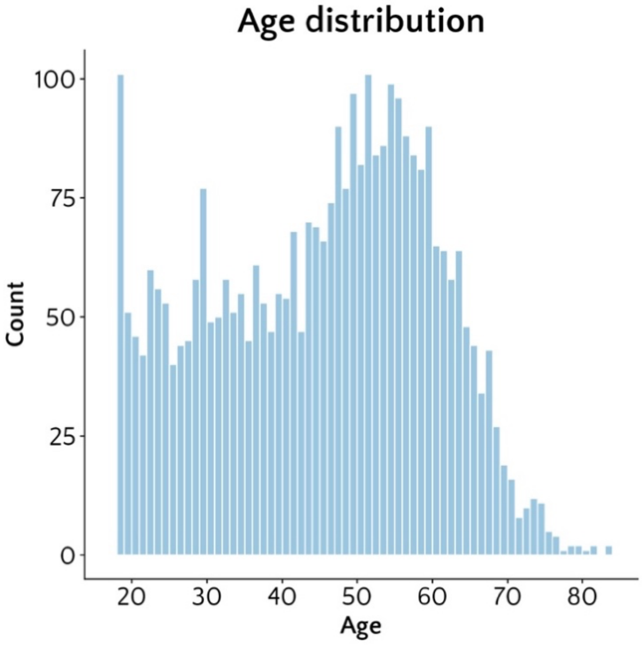

Figure S1. **Age histogram showing the number of participants per years of age in the final sample (N = 3,342).**

S2: Sensitivity analysis excluding non-native speakers

We have replicated the main analyses of the paper in a subset of n = 2,996 participants who reported having English as their first language. This analysis did not reveal a substantially different pattern of results, although the association between diabetes and working memory was rendered non-significant at *P* < .0038 (see Figure S2 below), probably due to the reduction in sample size. Additionally, the association between history of stroke and binarized cognitive flexibility (panel C) was now significant, unlike in the full sample analyses. Overall, these results illustrate that the inclusion of non-native speakers did not have a substantial effect on the results.

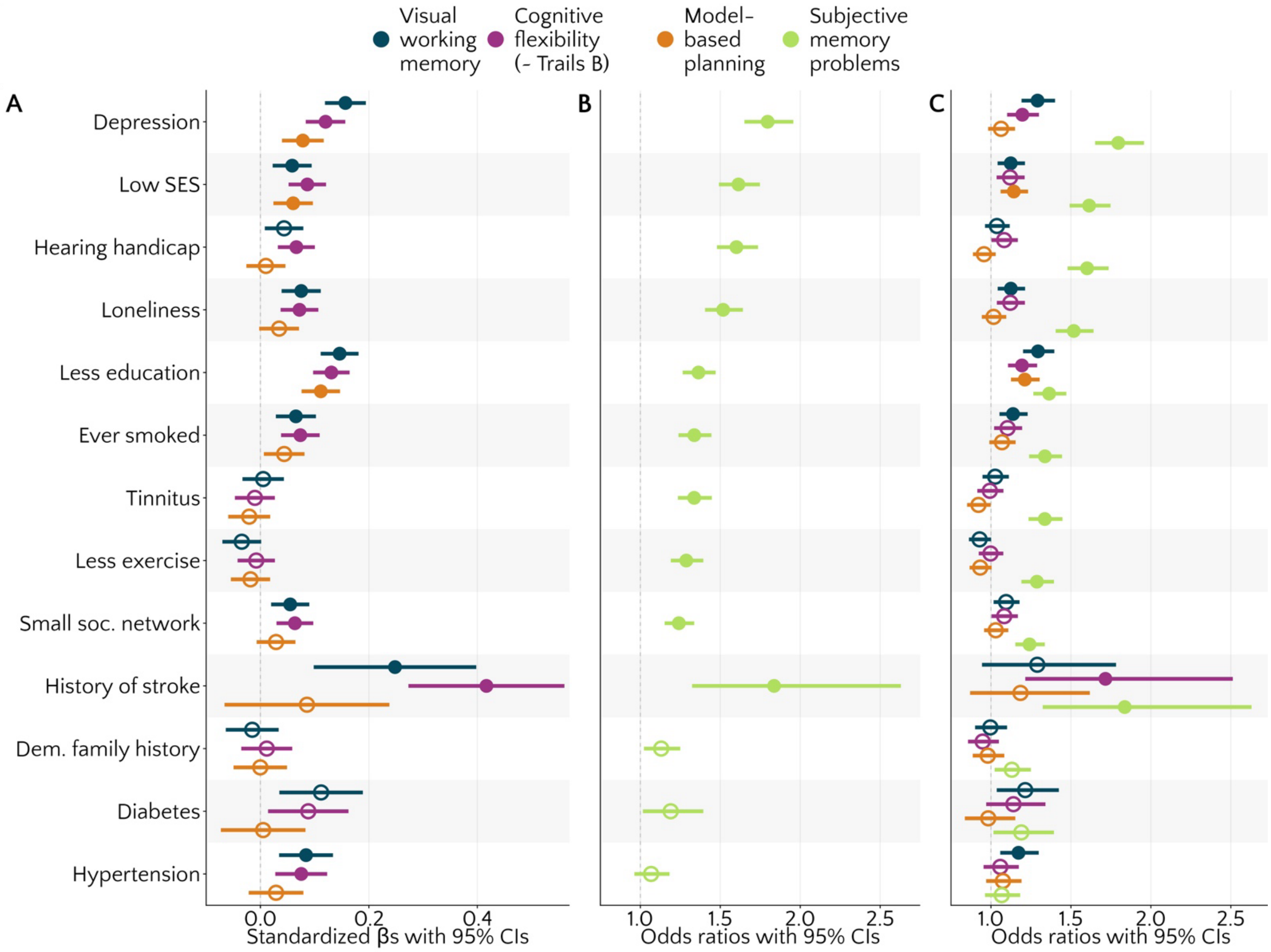

***Figure S2.* Associations between cognitive measures and risk factors, controlling for age and gender, in the subset of native-speaking participants (n = 2,996). Filled circles represent values significant at P < .0038, i.e., after applying Bonferroni correction per dependent variable. Higher estimates indicate worse cognitive outcomes. Circles and lines represent (A) standardized beta estimates with 95% confidence intervals or (B–C) odds ratios with 95% confidence intervals. In (C), objective cognitive scores were binarized to enable direct comparison of odds ratios with subjective memory problems.**

S3: Validation of gamified cognitive measures

We developed gamified smartphone-based versions of three cognitive tests that have been linked, directly or indirectly, to incipient cognitive decline and Alzheimer's dementia. In brief, compared to the traditional tests, our newly developed games take less time to complete, aim to be more engaging, and can be fully self-administered via smartphone while maintaining a standardized presentation of stimuli. A detailed validation of the task that assesses model-based planning 'Cannon Blast' has already been published[1] and the methodology and results are summarised briefly below. For the cognitive flexibility task ('Star Racer') and the visual short term memory task ('Memory Match'), we describe their validation here. In each case, we compared the new tasks with their traditional counterparts in an in-lab study and further assessed the validity and reliability in a large sample crowdsourced via the Neureka app.

S3A Memory Match and Star Racer

Method

Participants. For both Memory Match and Star Racer, we conducted a separate in-lab validation study, in which paid participants completed the two games together, in addition to more traditional versions of these tasks (Visual Short-Term Memory Binding Task – VSMBT,[2] and Trail-Making Test – TMT,[3] respectively). We recruited a convenience sample at Trinity College Dublin, Ireland, through posters on campus and online advertisements and from the Greater Dublin area via online advertisements in the Dublin volunteer centre. Of the originally recruited $N = 45$ participants, $n = 35$ had complete data for both Memory Match and VSMBT and $n = 41$ participants had complete data for both Star Racer and TMT. Further two participants were excluded from the Memory Match/VSMBT sub-sample – one due to colour-blindness and the other due to not passing a perceptual check (see Procedure, section Traditional binding task below). The final $n = 33$ Memory Match/VSMBT participants were aged 18–46 ($M = 24.7 \pm 7.1$) and consisted of 63 % female participants. The final $n = 41$ Star Racer/TMT participants were aged 18–68 ($M = 26.2 \pm 9.9$) and consisted of 56 % female participants.

Second, we used data crowdsourced from unpaid 'citizen scientists' (i.e., volunteer members of the general public) who have completed Memory Match or Star Racer within the 'Risk Factors Science Challenge' module in the Neureka app since the app's release in June 2020 until January 2023. After removing incomplete datasets, participants who left and re-entered the app during gameplay, and Star Racer response times that exceeded cut-off times (i.e., over 100s on Star Racer A and over 300s on Star Racer B, see more in the Materials section below), the final samples consisted of N = 6,398 (Memory Match), respective N = 5,986 (Star Racer) participants. Participants were aged 18–85 ($M = 45.2 \pm 14.7$ for Memory Match, respective $M = 44.5 \pm 14.7$ for Star Racer). With regards to gender, 4,161 (65.0%) for Memory Match, respective 3,841 (64.2%) for Star Racer were cisgender female, 107 (1.7%), respective 108 (1.8%) were non-cisgender, and 15 (0.2%), respective 18 (0.3%) preferred not

to state their gender. The participants came from 74 (Memory Match), respective 73 (Star Racer) countries, with the United Kingdom, United States, Ireland, Canada, and Germany being the most prevalent. Additionally, a subset of n = 294 (Memory Match), respective n = 67 (Star Racer) participants completed a part of the ‘Free Play’ module in the Neureka app that was comparable to the ‘Risk Factors’ version of each of the games, providing us with data for a test-retest reliability estimate.

Procedure.

Study 1 (in-lab validation study). Participants were administered Memory Match, Star Racer, and both traditional tasks in semi-randomized order: Each gamified task and its traditional version were administered together in blocks but the order of tasks within blocks and the order of blocks were both randomized. Both Memory Match and Star Racer were administered on a Google Pixel 3a phone. After completing the tasks, participants provided their demographic data, then they were debriefed and reimbursed.

Study 2 (citizen scientists). Both Memory Match and Star Racer were included in the ‘Risk Factors Science Challenge’ module in the Neureka app and administered remotely to volunteer citizen scientists alongside self-report lifestyle and health questionnaires. The Risk Factors challenge is further described in the main part of this paper. Importantly, the order of presentation of the tasks was randomized and completion of the entire challenge could be distributed across time. In the current analyses, we included the first complete take on each task (Memory Match /Star Racer) in the Risk Factors challenge. Additionally, modified versions of both Memory Match and Star Racer were available in the ‘Free Play’ section of the Neureka app as standalone games, where the participants could choose which difficulty level of each game they complete and how many times. We used performance on completed Free Play takes and compared them to Risk Factors takes to estimate test-retest reliability of each task. Both studies were approved by the Trinity College Dublin School of Psychology Ethics Committee and informed consent was obtained from all participants prior to participation.

Materials.

Traditional binding task. The Visual Short-Term Memory Binding Task (VSMBT) is a computer-based task that uses a change-detection paradigm to assess visual working memory binding (i.e. the ability to associate multiple features together in short-term memory), which seems to be specifically impaired early in Alzheimer’s disease but not in normal ageing.[2,4–6] In our study, VSMBT was administered using E-Prime 2.0 Run Time.[7] Like Brockmole et. al,[4] we used an array of black shapes (non-binding condition) or coloured shapes (binding condition) as stimuli. The shapes on each trial were randomly selected out of a set of eight six-sided polygons and presented on light grey background. The colours in the binding condition were randomly selected from a set of eight non-primary colours. No shape or colour was repeated within any given array. On each trial, a study array was presented followed by a test array, whereby the participants had to recognize whether the items

presented in the test and study arrays were the ‘same’ or ‘different’ and to respond accordingly by a button press (see Figure S3 for a detailed overview of the task). On the ‘different’ trials, studied shapes were replaced by new ones (non-binding condition), or two shapes swapped colours (binding condition). Location of the stimuli changed randomly between study and test arrays to prevent participants from using location as a memory cue.

The task in the current study consisted of four conditions, with two-item and three-item versions of both the non-binding and the binding condition. There were 16 ‘same’ trials and 16 ‘different’ trials in each condition, presented in random order, and so participants completed 128 trials in total. Conditions were blocked: All participants first completed a two-item block of the non-binding condition, followed by a two-item block of the binding condition. They were then shown two blocks of three-item displays, with half of participants receiving the non-binding block first. The order of conditions (non-binding or binding first) for the three-item blocks alternated between participants. To ensure that the participants understood the task, the examiner showed them sample arrays, gave an oral explanation of the task, and asked for their response (‘same’ or ‘different’), before each block started.

Between the two-item and three-item blocks of trials, participants additionally completed a brief perceptual task to check if their performance could be confounded by perceptual problems independent of memory. In each of the 10 trials of this perceptual task, participants were presented with two sets of three coloured shapes, which appeared simultaneously on-screen separated by a horizontal line. Participants were asked to indicate whether the two sets of shapes were the same or different, with five ‘same’ and five ‘different’ trials being ordered randomly across task. Scoring under 90% correct (less than 9 out of 10 trials) was considered indicative of perceptual binding difficulties, resulting in one participant being excluded from the study.

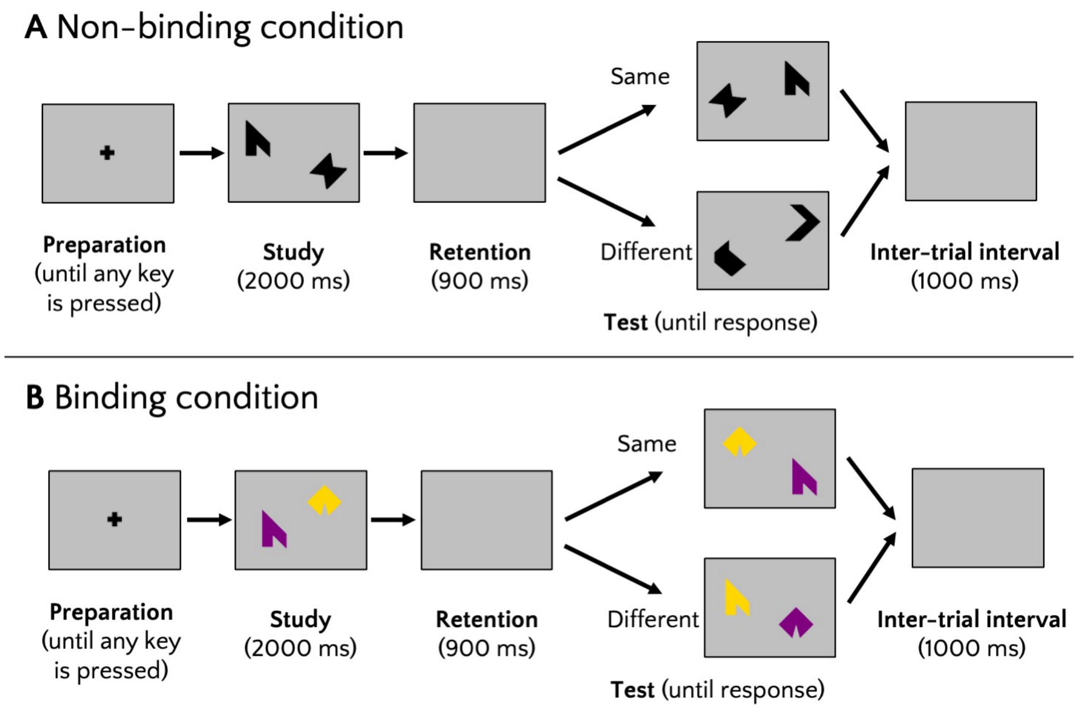

***Figure S3.* Traditional binding task (VSMBT), two-item version. A: The non-binding condition, in which participants only need to memorise the shapes. B: The binding condition, in which participants need to memorise shape-colour combinations. Note that in the 'different' trials, test set shapes are the same as in the study set but swap colours.**

Memory Match. Besides the Risk Factors challenge (see main paper for more information), Memory Match was also available in a modified 'Free Play' version. In this version of the task within a separate section of the app, each of the difficulty levels was available for playing individually. The task setup within each difficulty level was otherwise identical. Unlike in the Risk Factors version, the self-paced task instructions (Supplement S4, Figure S8A) were not presented automatically at the start of each gameplay, but they were available for re-visiting individually within the 'Free Play' section.

Traditional trail-making test. The Trail Making Test (TMT) is a pen-and-paper cognitive test, commonly used to detect neuropsychological impairment both in research and clinical settings.[3] It consists of two parts that require the participant to connect 25 labelled circles in numerical order (TMT A) or numerical and alphabetical order, alternating between numbers and letters (i.e., 1–A–2–B–etc.; TMT B). The main variable of interest is the total time to complete each part. TMT A taps mainly into processing speed and visual search, whereas TMT B taps mainly into cognitive flexibility and executive function.[3] We administered the traditional trail-making test according to standard instructions[3] – including two practice runs and both A and B versions.

Star Racer. Participants can play Star Racer within the Risk Factors challenge or in a 'Free Play' section of the app. In Risk Factors, where most participants first encounter the game, it begins with a set of self-paced tutorial screens and practice runs (see Supplement S5, Figure S9A). In this tutorial, participants receive static screens illustrating the task and then

complete a short practice round of trails A and B, with just eight stars. Once the participants tap 'Start Game', this is followed by six runs of the main task (see Supplement S5, Figure S9B). Of note, the first two runs of A and B mirror closely the original layout of A and B forms and are as such are referred to as "hard-coded". The remaining 4 runs have randomly generated star locations (i.e., "random-coded"), thus setting up the task for repeated administration with reduced learning effects.

In Free Play, there are three difficulty levels that participants can choose from – two easier levels with fewer stars ('Easy' with 8 stars and 'Medium' with 15 stars) and the third one with the same number of stars as in the Risk Factors version of Star Racer ('Hard', 25 stars). The 'Hard' level consists of one run of each version A or B, with randomly generated star locations. Unlike in Risk Factors, the task instructions and practice runs are not presented automatically at the start of gameplay, but they are available for re-visiting within the 'Free Play' section.

In both the Risk Factors and the Free Play version of the task, we applied exclusion criteria as specified in the main paper to deal with inattentive responders: We excluded all runs of version A that exceeded 100 seconds, and all runs of version B that exceeded 300 seconds. Performance was calculated as the mean time to complete the remaining runs for each participant. These cut-offs were based on approximately double the median completion times for the oldest and least educated group in a normative study of the traditional task.[8]

Analyses.

Validity. In the in-lab validation study sample, we estimated convergent validity by calculating correlations of each of the new tasks and its different conditions with more traditional versions. We also ran an ANOVA with Tukey's post-hoc tests (Memory Match) and a series of two-sample t-tests (Star Racer) as applicable across both samples (i.e., in-lab and citizen scientist) to compare each task's conditions among themselves and establish the extent to which each task behaves similarly to its traditional version. To assess the ceiling effects in Memory Match and VSMBT, we used a paired t-test to compare mean performance and a Levene's test to compare variability of both tasks in the in-lab sample.

Internal consistency. Split-half reliability of Memory Match was calculated using the Pearson correlation across odd and even trials in the large Citizen Science sample. To generate a comparison, we also calculated split half of the VSMBT from the in-lab validation study sample. Due to the task version we used, we did not have access to trial level data (only block-level), and so split half was calculated as the correlation of mean accuracy across the trials with 2 stimuli to remember vs those with 3 stimuli to remember. To adjust for test length effects, Spearman–Brown formula was applied to the resultant correlations. For Star Racer, internal consistency was calculated for A and B versions separately as Cronbach's α of the three runs in each version, using the data from the citizen scientists. We did not have a comparator for the traditional task, as it is a paper and pen assessment with only one measure

of total time to complete. Additionally, we computed bootstrap 95% confidence intervals for Cronbach's α based on 1000 samples.

Test-retest reliability. We calculated test-retest reliability as a Pearson correlation between overall performance on Memory Match and Star Racer (separately for the A and B versions) in the Risk Factors section and the equivalent subset of Free Play data. We only included participants who completed the Risk Factors and the Free Play versions of the games within 30 days from each other. The median distance between assessments was 1 day for both Memory Match and Star Racer. As the Free Play version of each game had fewer trials/runs compared to the full Risk Factors version and participants could complete the various difficulty levels in whatever order they preferred, we only used data from participants who completed trials/runs in a number and difficulty that was equivalent to the Risk Factors version of each of the games, and only if they completed all these equivalent Free Play trials/runs within the same day. For Memory Match, overall accuracy (i.e., mean proportion correct) of each participant was calculated by averaging the proportion correct from their first attempt at each difficulty level in the Free Play. For Star Racer, mean completion time of each participant was calculated by averaging completion time on their first 3 attempts at the 'Hard' difficulty level in the Free Play section.

Results

Memory Match. We found that as expected, the overall accuracy on Memory Match decreased as memory load (i.e., number of memorised items) increased (F (2) = 7729.17, P < .001, η^2 = .15; see Figure S4A). Performance on Memory Match also differed by condition (F (1) = 1476.38, P < .001, η^2 = .02; see Figure S4B) and trial type (F (1) = 52677.82, P < .001, η^2 = .38), whereby it decreased as task conditions became more complex (i.e., abstract shapes < nameable letters, binding < non-binding; all P < .001), mirroring the effects seen in previous VSMBT literature. Importantly, we were successful in reducing ceiling effects; the mean overall performance was significantly lower (t (32) = 7.77; P < .001) and variability higher (F (1, 64) = 16.38; P < .001) on Memory Match (M = .83 ± 0.08) than on VSMBT (M = .94 ± .04). Given this important enhancement to the task (see ranges of scores in Figure S4D, S4E), we did not expect perfect cross-task convergence. In the in-lab sample, the correlation between overall accuracy on Memory Match and VSMBT was r (31) = .40; P = 0.023 (Figure S4D). For Memory Match, the test-retest reliability of overall accuracy was assessed in those citizen scientists who played the game more than once and we found moderate reliability, r (292) = .63, P < .001. The split-half reliability of overall Memory Match accuracy was assessed in the larger sample who completed the game once, giving r (6396) = .64, P < .001 (r = .78 after adjusting for test-length effects using the Spearman-Brown formula), suggesting an acceptable internal consistency. These reliability estimates are comparable to the traditional version of the task where the correlation across the two levels of the task was r (31) = .60 (r = .75 after adjusting for test-length effects using the Spearman-Brown formula).

One point of departure across the traditional and this new smartphone test concerned the binding effect[4] – while we replicated the overall binding effect (i.e., mean accuracy lower

on binding vs non-binding trials; Figure S4B) on aggregated performance, we noted that this was driven by the ‘letters’ trials in Memory Match. In the ‘shapes’ trials, participants showed no difference (in-lab sample) or even performed slightly worse (citizen scientists) on the non-binding than the binding condition (Figure S4C). This was reflected in a significant interaction effect of condition x trial type in citizen scientists (F (1) = 2936.44, P < .001, η^2 = .03). We suspect this is a feature of the use of a grid-search design instead of the traditional task’s forced-choice paradigm where participants have to categorize presented symbols as ‘same/different’. Because the target shapes are particularly difficult to identify within a grid of many other abstract shapes, on binding trials, it is possible participants utilized colour information to narrow down the number of shapes they had to consider and boost performance that way (see Figure S5). We found a comparable pattern of results in the smaller, in-lab sample (see Figure S4A–C). Overall, though interesting, as we focus on overall working memory performance, these details do not affect the key interpretation of our dependent measure.

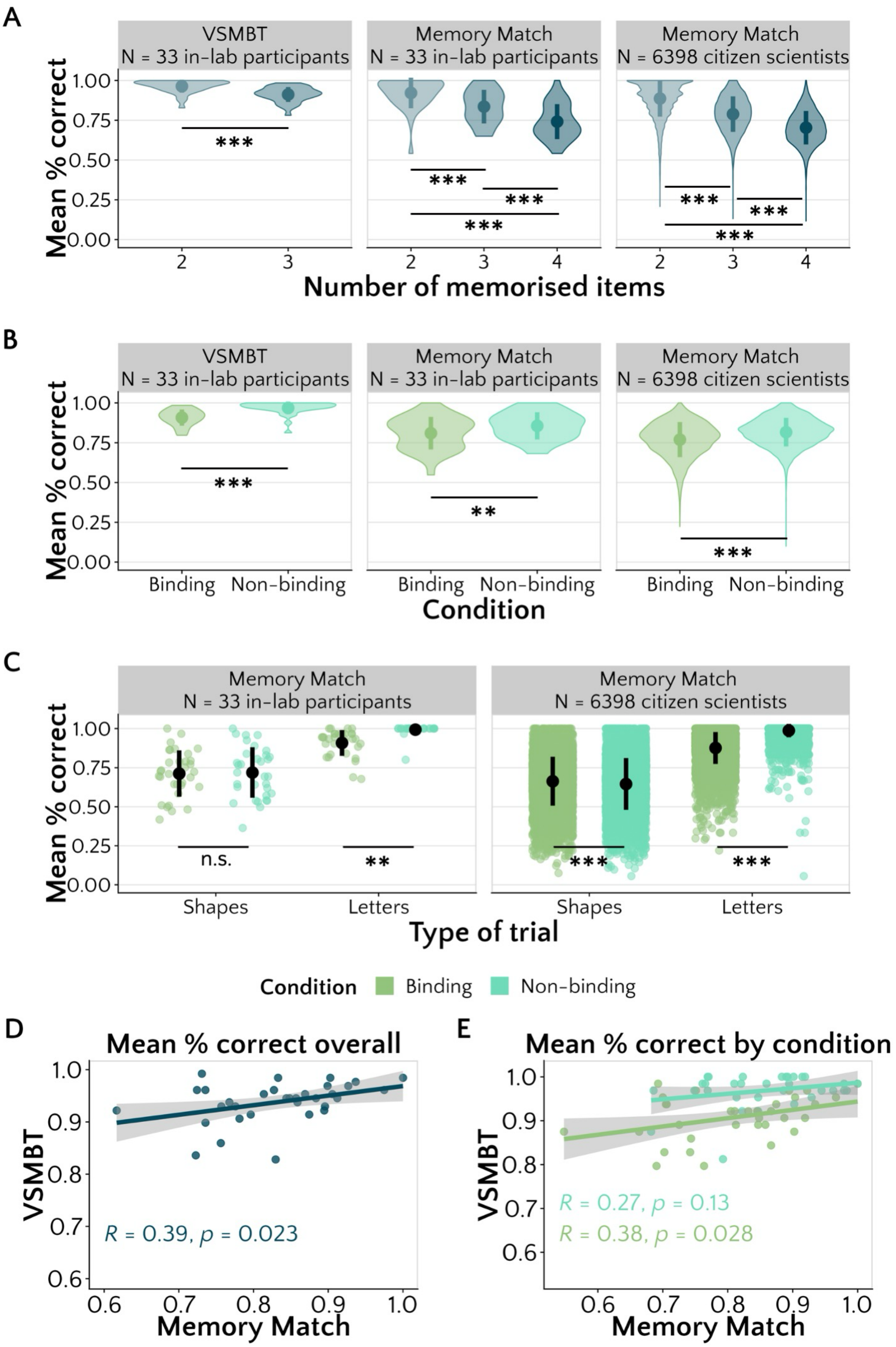

Figure S4. **A: Mean percentage correct in VSMBT and Memory Match, split by difficulty level (i.e., number of memorised items). B: Mean percentage correct in VSMBT and Memory Match, split by condition (i.e., binding/non-binding). C: Mean percentage correct in VSMBT and Memory Match, split by condition and trial type (i.e., shapes/letters). D: Correlation of mean percentage correct (i.e., overall accuracy) in VSMBT and in Memory Match. E: Correlation of mean percentage correct in VSMBT and in Memory Match, split by condition.**

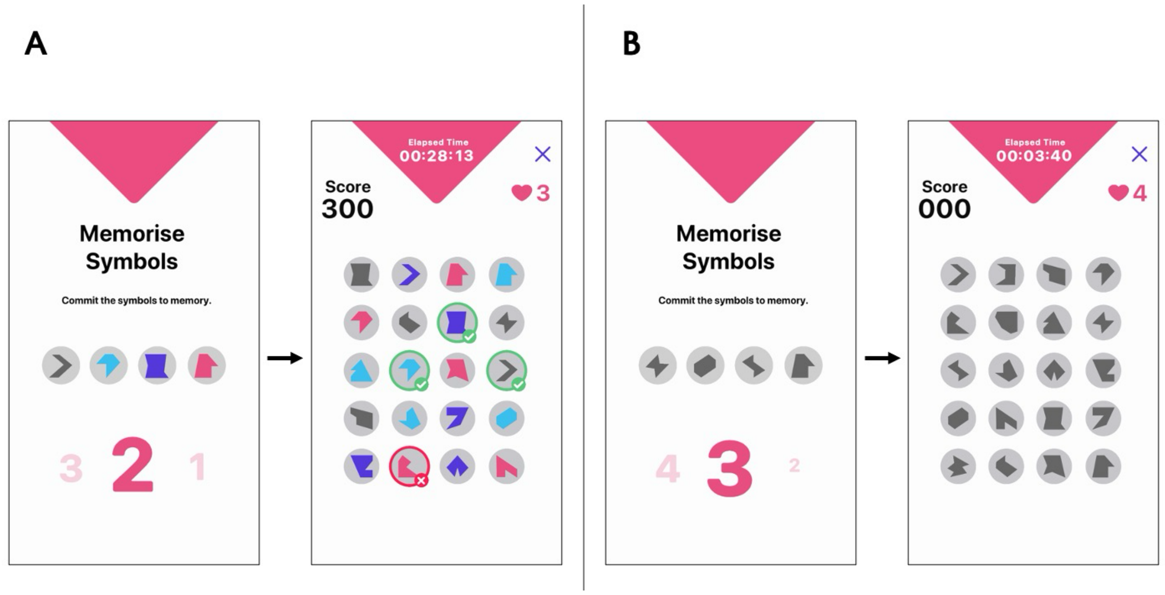

Figure S5. **Comparison of the binding (A) and non-binding (B) condition of a shapes trial. The colour in the binding condition can serve as guidance for participants, whereby in comparison to the non-binding task, they have a reduced set of shapes of each colour to choose from.**

Star Racer. Similarly to the traditional TMT (t (40) = -12.66, P < .001), completion times in Star Racer were larger in version B compared to version A consistently across task conditions (Figure S6A) – both in runs with hard-coded (t (40) = -12.84, P < .001) and random star positions (t (40) = -8.86, P < .001) of the Risk Factors version, as well as the Free Play version of Star Racer (i.e., always random star positions; t (1539) =-55.935, P < .001). In the in-lab validation sample, performance on Star Racer A or B was correlated with completion time on the corresponding A or B version of TMT – both when split by condition (Figure S6B), as well as when taking into account mean completion time across all 3 runs of Star Racer A (r (39) = .47, P = .002) and Star Racer B (r (39) = .63, P < .001; see Figure S6C). Cronbach's alpha was α [95% CI] = .82 [.81, .83] for Star Racer A and .77 [.76, .79] for Star Racer B, suggesting acceptable to good internal consistency. The test-retest reliability of mean completion time was good to moderate (r (65) = .87, P < .001 for Star Racer A, r (66) = .72, P < .001 for Star Racer B).

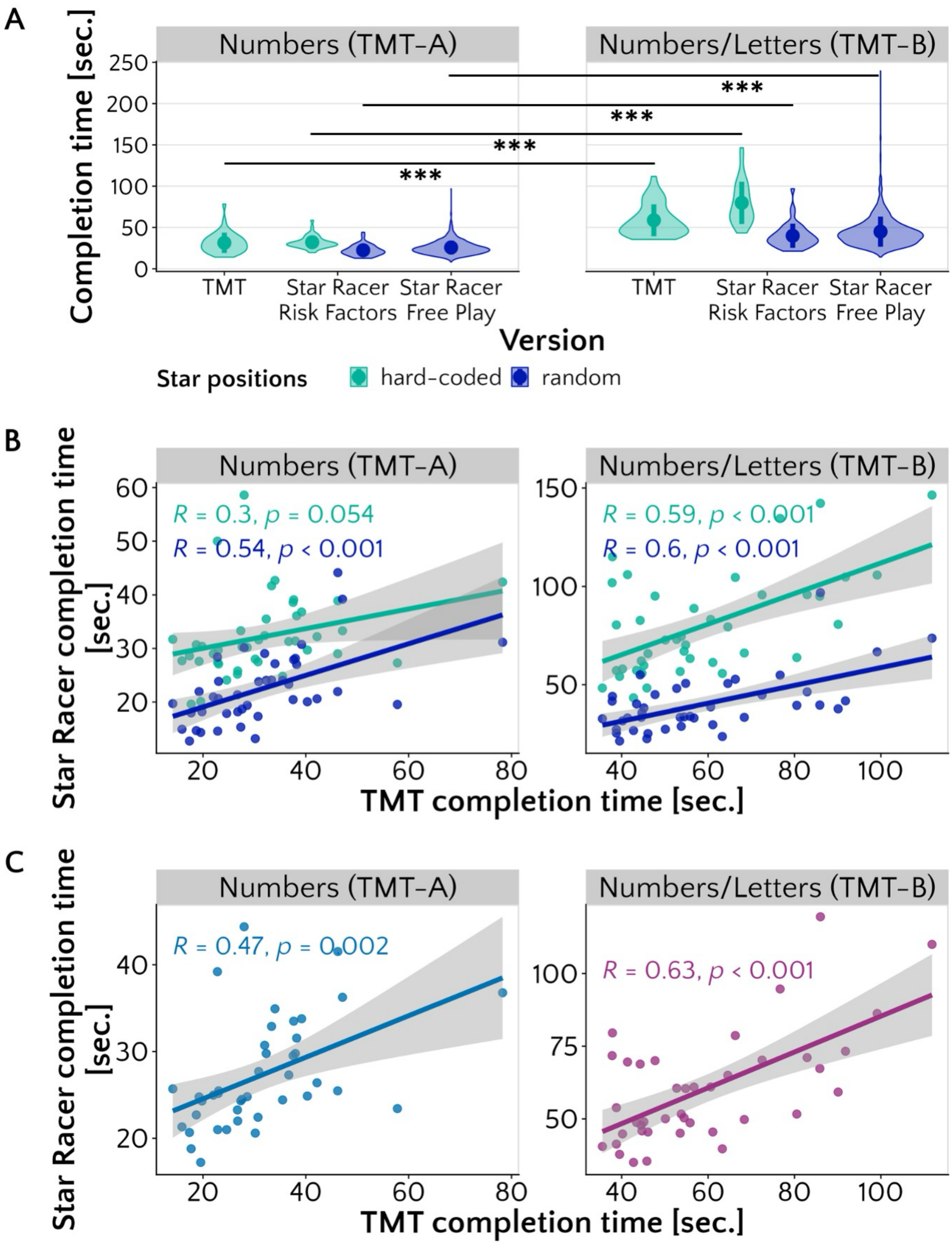

***Figure S6*. A: Comparison of completion times on versions A and B in hard difficulty level (25 stars), from left to right: (i) TMT, in-lab sample; (ii) Star Racer in Risk Factors, only runs with hard-coded star positions (i.e., layout directly comparable to TMT), in-lab sample; (iii) Star Racer in Risk Factors, average performance across two runs with random star positions, in-lab sample; (iv) Star Racer in Free Play (where all runs had random star positions), citizen scientist sample. B: Correlation between Star Racer and TMT completion times, split by version (A/B) and trial type (hard-coded/random star positions). C: Correlation between mean completion time in the Risk Factors version of Star Racer and TMT completion time, split by version (A/B).**

S3B Cannon Blast

Cannon Blast is a gamified version of the 'Two-Step Reinforcement Learning Task'.[9] It includes key elements of the original task's structure (i.e., drifting rewards, a probabilistic transition structure), wrapped up in diamond shooting game (see Figure S7). In this game, users attempt to strike diamonds, which are sometimes moving around the screen or partially obstructed, by firing from one of two containers on the screen. Each container has a mix of purple and pink balls; one has 80% pink balls and the other 80% purple, corresponding directly to the probability that a ball of that colour will be released – this is what we refer to as 'task structure'. This means that someone can intentionally choose to increase their chances of firing a pink or purple ball. Crucially, in this task, some balls explode upon firing and therefore cannot reach their target. This is not at random, but rather is partially predictable from the colour of the ball, whereby the chances that a pink/purple ball will explode drifts slowly and independently over the course of the task. This means that a person can reduce their chances of getting a bad ball by tracking which ball is currently bad and choosing the container least likely to produce it. This is the signature of model-based planning on the task. To operationalise this, data were analysed using a well-established procedure – using hierarchical logistic regression (HLR) models, which are mixed effects models implemented with the lme4 package in R.[10] The model tests if participants' choice behaviour in the first stage state (coded as switch: 0 and stay: 1, relative to their previous choice) was influenced by IVs tracking (i) whether that ball was good or bad on the last trial (coded as bad: -1 and good:1) and (ii) whether the last trial was a trial where the ball produced from the container was the one expected by the explicit probability ('transition' coded as uncommon: -1 and common: 1), and (iii) their interaction. Within-participants factors (main effect of reward, transition and their interaction) were modelled as random effects. Model-based index (MBI) is quantified as the interaction between Reward (good vs. dud ball) and Transition (common vs uncommon ball colour appearing from the chosen container). Individual estimates of the MBI were extracted and a single value for each participant was brought forward for the main analysis.

A prior paper validated this task in detail.[1] In brief, there was a moderate positive association between MBI derived from *Cannon Blast* and the Traditional task ($r = .40$, $P = .002$). Split-half reliability for MBI were high similar for both the traditional ($r = .81$, 95% CI [.70, .88], $P < .001$) and *Cannon Blast* ($r = .78$, 95% CI [.66, .87], $P < .001$). The test-retest reliability was r (423) = .63 assessed over a variable interval (median 4 days).

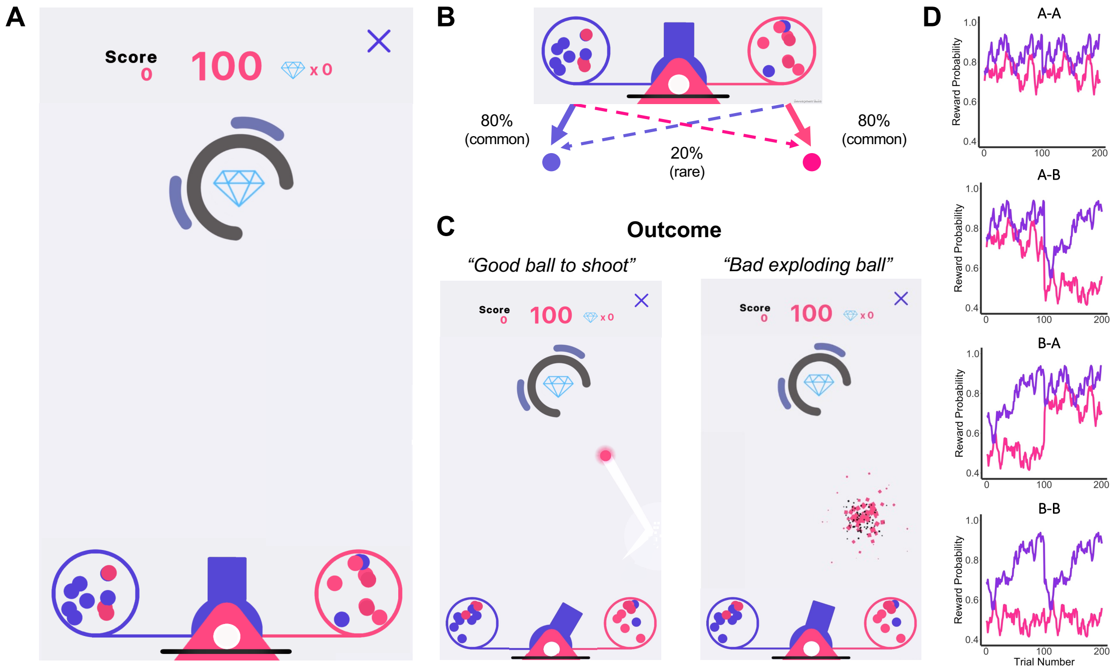

Figure S7. **Task structure of *Cannon Blast*, a smartphone game to assess model-based planning. A. In this game, participants’ goal is to shoot as many diamonds as possible before their total number of shots (100 per block) runs out. To do so, they must aim a central cannon and then select which circular container to draw from. B. Purple and pink balls dynamically bounce around each of the flanked containers which depict the probability of a pink or purple ball being released. For example, the left container displays 8 purple balls and releases a purple ball 80% of the time (‘common’ transition) and displays 2 pink balls, giving a pink ball on 20% of trials (‘rare’ transition). C. The purple and pink balls have different values that dynamically change throughout the game. The value of the ball is defined as the probability of it being a ‘good ball’, i.e., one that remains intact after firing (rewarding trial), or a ‘dud ball’ (non-rewarding trial) that explodes shortly after being fired, and therefore cannot reach the diamond. D. We included 2 drifting reward probabilities (A, B) that quantitively differed on various metrics. Participants were randomly assigned a reward drift set at each block leading to four distinct drift set combinations (A-A, A-B, B-A, B-B). Figure and legend reproduced with permission from Donegan et al.,[1] published in Communications Psychology under the CC BY 4.0 licence (https://creativecommons.org/licenses/by/4.0/).**

S4: Task structure of Memory Match

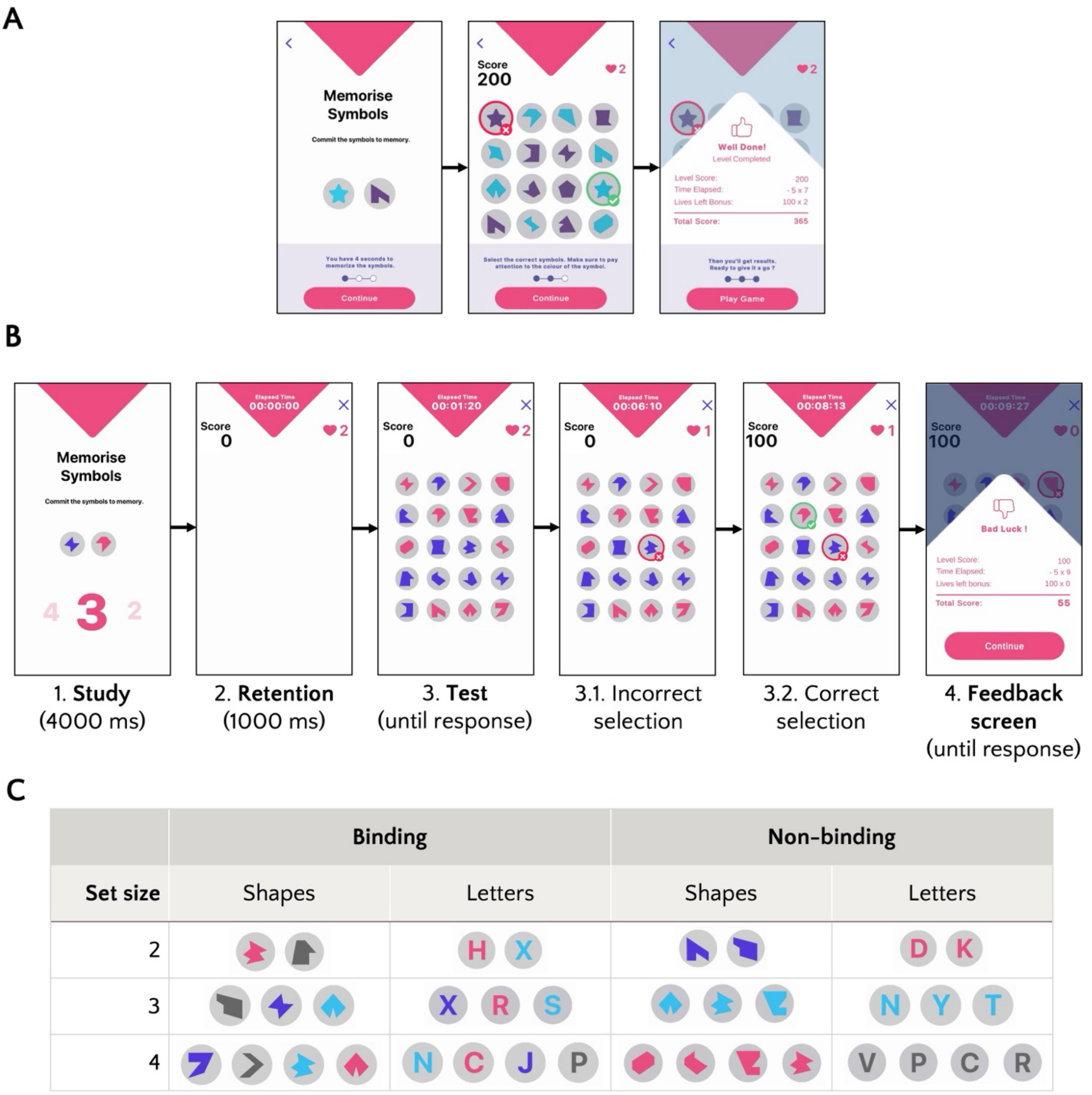

Figure S8. A: Self-paced task instruction screens that appear at the start of Memory Match within the Risk Factors challenge. B: An example trial of Memory Match. The presentation of the study array (1.) is followed by a retention interval (2.) until the test display (3.) fully loads. Participants can lose lives and points by making incorrect selections (3.1.) or earn points by making correct selections (3.2.). Each trial is concluded by a feedback screen (4.). C: Examples of stimuli used in different trial types of Memory Match.

S5: Task structure of Star Racer

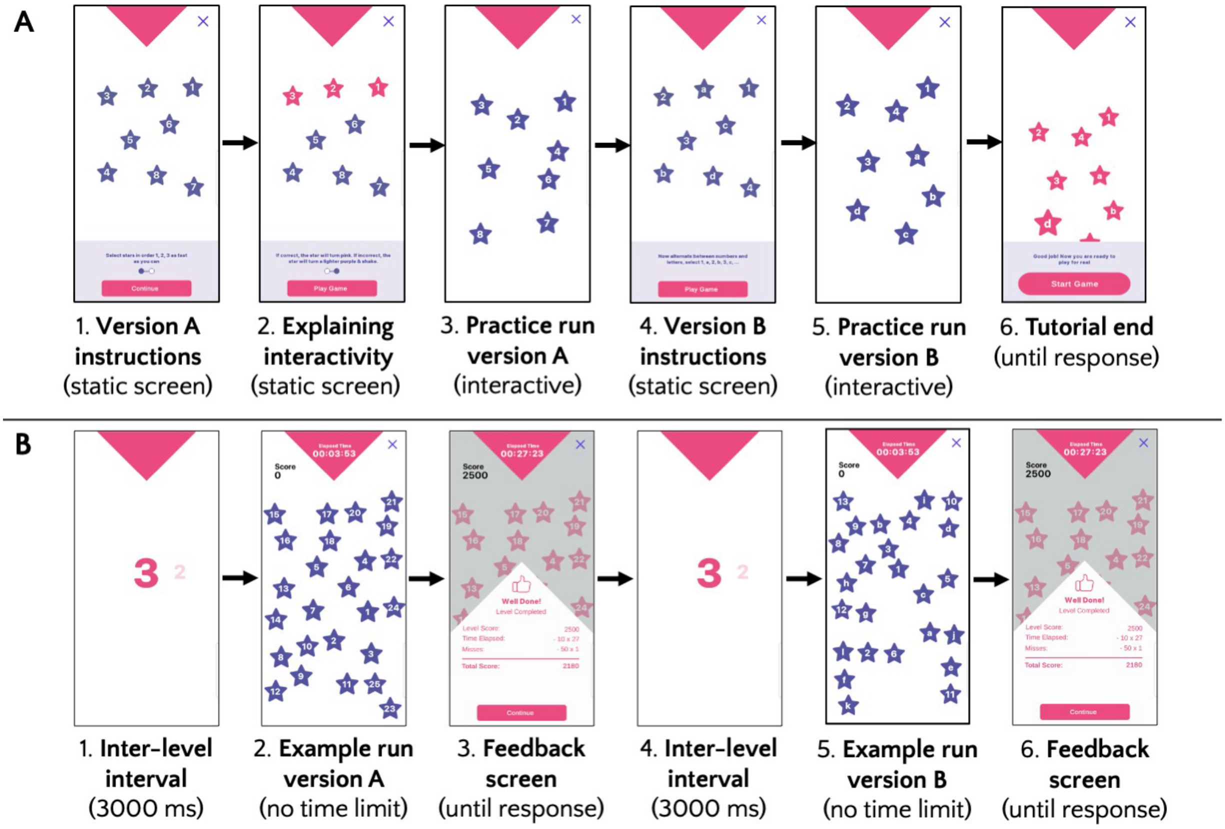

Figure S9. **A: Self-paced instruction screens and practice runs that appear at the start of Star Racer within the Risk Factors challenge. B: Example of Star Racer runs (first A, then B version) with hard-coded star positions.**

S6: Associations between risk factor measures

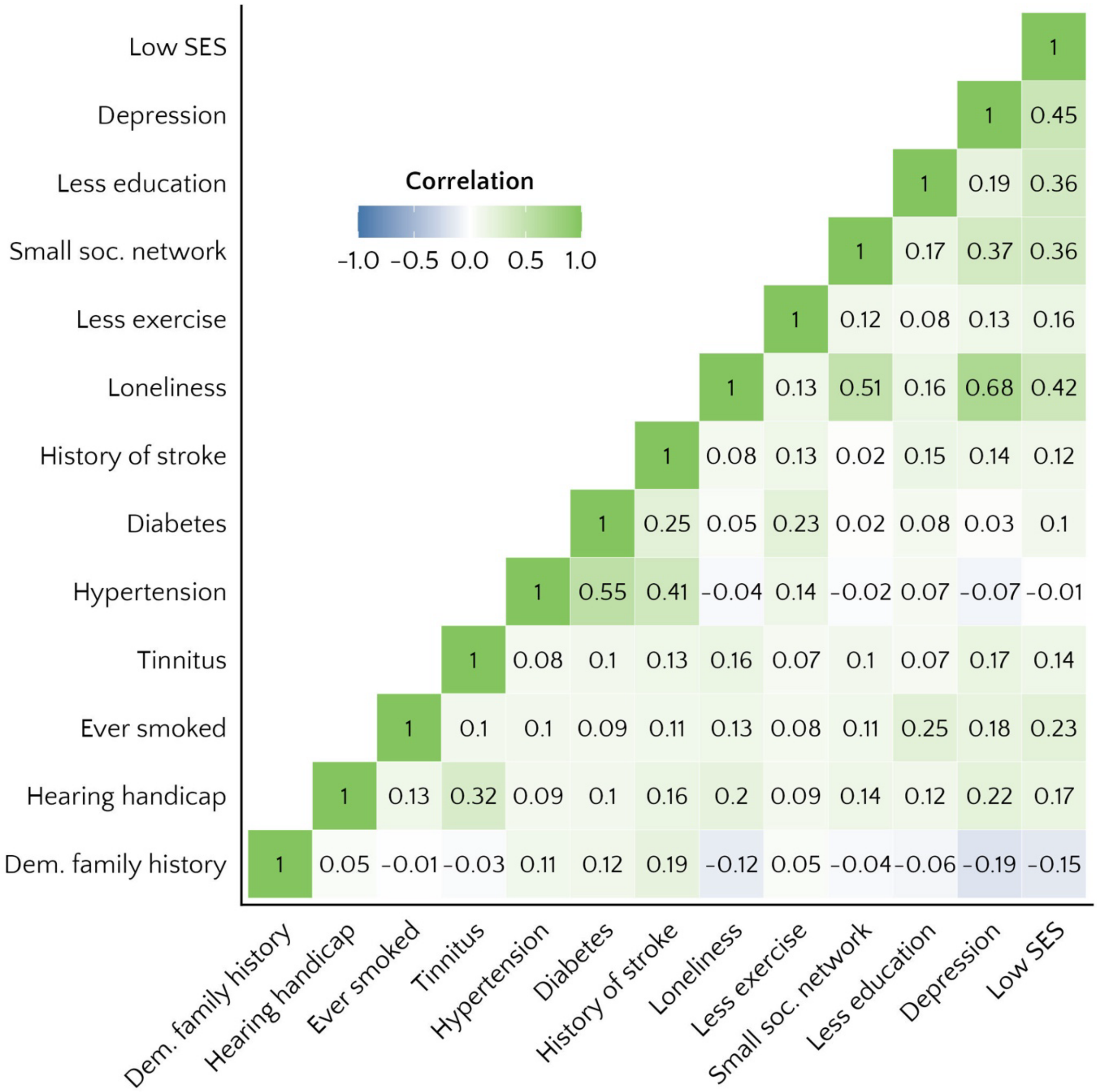

Figure S10. **Correlation matrix of all biopsychosocial factors assessed in the current study (calculated in the final sample, N = 3,342). Numbers represent Pearson product-moment correlation coefficients for pairs of continuous variables, polyserial correlations for pairs of continuous and categorical variables, and polychoric correlations for pairs of categorical variables.**

S7: Results for non-cisgender participants

Compared to cisgender men, non-cisgender individuals had significantly better visual working memory (β [95% CI] = -0.21 [-0.34, -0.09]; P = .001; see Figure S11A), cognitive flexibility (β [95% CI] = -0.16 [-0.28, -0.04]; P = .008; see Figure S11B), and model-based planning (β [95% CI] = -0.14 [-0.26, -0.02]; P = .028; see Figure S11C), but also a significantly higher likelihood to report subjective memory problems (OR [95% CI] = 1.64, [1.28, 2.12], P < .001; see Figure S11D).

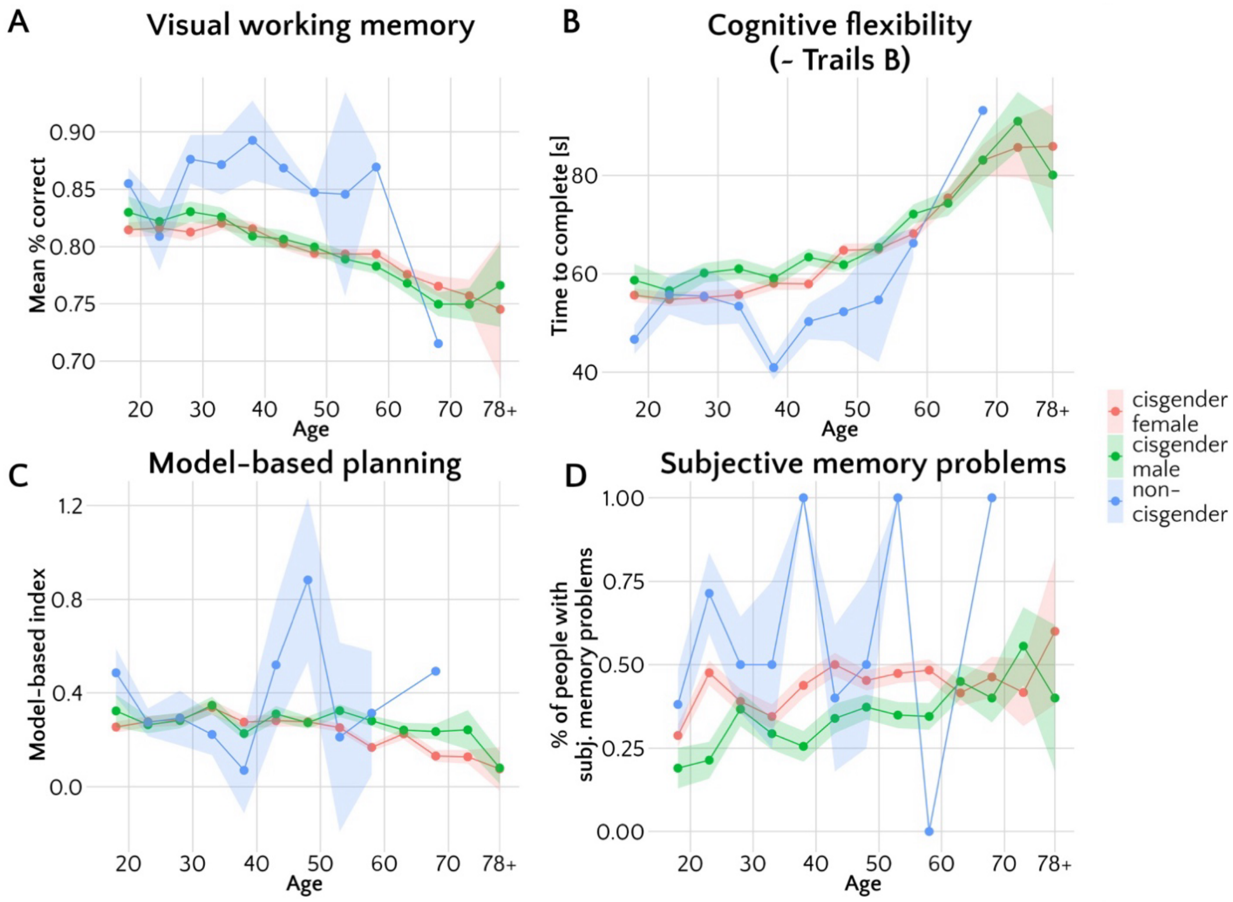

Figure S11. **Associations of cognitive measures with age and gender, including cisgender females and males and "non-cisgender" participants (i.e., participants who identified as either non-binary, transgender female, or transgender male). Points correspond to mean raw scores on (A) visual working memory, (B) cognitive flexibility, (C) model-based planning, and (D) mean proportion of participants with memory problems. The means were calculated per 5-year bins, split by gender. Error bars represent standard errors (A–C) or standard errors of proportion (D). Note that due to the very small number of non-cisgender participants (n = 67, i.e., 2% of the total sample), not all age groups were represented and calculating means or standard errors was not always possible**

S8: Comparison of effect magnitude for subjective vs. objective cognition

Table S1.

Associations of risk factors with binarized cognitive outcomes (n = 3,327), controlling for gender and age, expressed as estimates from logistic regressions for all DVs. Highlights indicate results significant after Bonferroni correction (P < 0.0038).

Risk factor	**Visual working memory**		**Cognitive flexibility (~ Trails B)**		**Model-based planning**		**Subjective memory problems**	
	OR [CI95%]	*P*	OR [CI95%]	*P*	OR [CI95%]	*P*	OR [CI95%]	*P*
Depression	**1.28 [1.18, 1.38]**	**< .001**	**1.16 [1.07, 1.25]**	**< .001**	1.05 [0.98, 1.14]	.166	**1.82 [1.68, 1.97]**	**< .001**
Low SES	**1.14 [1.06, 1.23]**	**< .001**	1.11 [1.03, 1.2]	.005	1.11 [1.03, 1.19]	.005	**1.65 [1.53, 1.78]**	**< .001**
Hearing handicap	1.04 [0.97, 1.11]	.327	1.07 [0.99, 1.15]	.068	0.96 [0.89, 1.03]	.236	**1.59 [1.47, 1.71]**	**< .001**
Loneliness	**1.12 [1.04, 1.2]**	**.003**	1.09 [1.01, 1.17]	.028	1.02 [0.95, 1.1]	.500	**1.52 [1.41, 1.64]**	**< .001**
Less education	**1.33 [1.24, 1.43]**	**< .001**	**1.18 [1.09, 1.27]**	**< .001**	**1.2 [1.12, 1.29]**	**< .001**	**1.39 [1.3, 1.5]**	**< .001**
Less exercise	1.03 [0.96, 1.11]	.357	1.06 [0.98, 1.14]	.147	1.01 [0.94, 1.08]	.761	**1.41 [1.31, 1.52]**	**< .001**
Ever smoked	**1.14 [1.05, 1.22]**	**.001**	1.08 [1, 1.17]	.039	1.07 [1, 1.15]	.067	**1.33 [1.24, 1.44]**	**< .001**
Tinnitus	1.02 [0.94, 1.1]	.609	1 [0.93, 1.08]	.971	0.92 [0.85, 0.99]	.037	**1.33 [1.23, 1.43]**	**< .001**
Small soc. network	1.1 [1.02, 1.18]	.012	1.08 [1.01, 1.16]	.032	1.04 [0.97, 1.12]	.232	**1.27 [1.18, 1.36]**	**< .001**
History of stroke	1.32 [0.98, 1.81]	.074	1.63 [1.18, 2.33]	.004	1.08 [0.8, 1.45]	.614	**1.84 [1.34, 2.6]**	**< .001**
Dem. family history	0.98 [0.89, 1.08]	.663	0.95 [0.86, 1.05]	.305	0.99 [0.9, 1.09]	.893	1.12 [1.01, 1.23]	.024
Diabetes	1.21 [1.04, 1.42]	.014	1.16 [0.99, 1.35]	.072	0.97 [0.83, 1.13]	.678	1.18 [1.01, 1.38]	.035
Hypertensio n	**1.17 [1.06, 1.29]**	**.002**	1.06 [0.96, 1.17]	.244	1.06 [0.96, 1.18]	.217	1.08 [0.97, 1.19]	.146

S9: Smoking history x age interaction

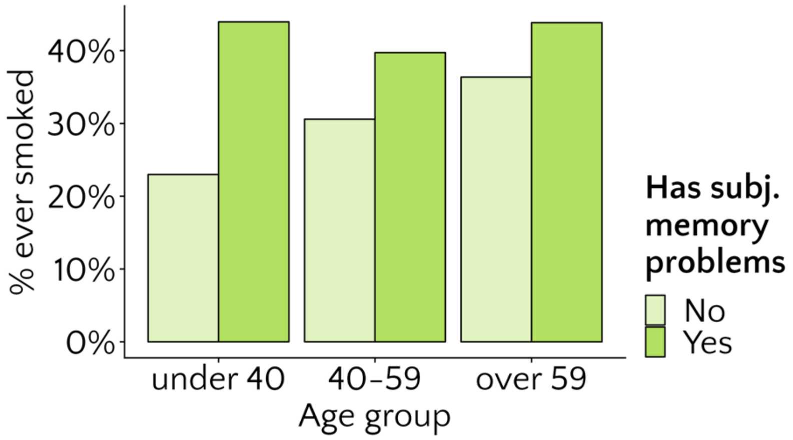

Figure S12. **Associations of smoking with subjective memory problems modified by age. For plotting purposes, age was grouped into three categories: young adults (under 40; n = 1,563), middle aged adults (40–59; n = 1,136), and older adults (over 59; n = 628).**

S10: Interactions between age and risk factors

Table S2.

Interactions of risk factors with age, regressed on cognitive outcomes, controlling for gender and age (n = 3,327). Estimates come from logistic regressions (subj. memory problems) or linear regressions (all other DVs). Highlights indicate significance at P < 0.0015.

Cognitive outcome	Interaction	*β (SE)*	*t/z*	*P*	OR [95% CI]
Visual working memory	Age * Small soc. network	-0.04 (0.02)	-2.50	.013	N/A
	Age * Low SES	-0.04 (0.02)	-2.27	.023	N/A
	Age * Loneliness	-0.05 (0.02)	-2.63	.009	N/A
	Age * Less education	-0.02 (0.02)	-1.34	.180	N/A
	Age * Hypertension	0 (0.03)	-0.06	.950	N/A
	Age * History of stroke	-0.13 (0.07)	-1.92	.055	N/A
	Age * Ever smoked	0 (0.02)	-0.15	.879	N/A
	Age * Diabetes	-0.09 (0.05)	-1.74	.083	N/A
	Age * Depression	-0.02 (0.02)	-1.17	.241	N/A
Cognitive flexibility (~ Trails B)	Age * Small soc. network	-0.02 (0.02)	-1.22	.221	N/A
	Age * Low SES	0 (0.02)	0.20	.839	N/A
	Age * Loneliness	-0.04 (0.02)	-2.40	.016	N/A
	Age * Less education	-0.02 (0.02)	-0.93	.350	N/A
	Age * Hypertension	0.06 (0.03)	2.08	.037	N/A
	Age * History of stroke	-0.08 (0.07)	-1.17	.244	N/A
	Age * Hearing handicap	0.01 (0.02)	0.40	.687	N/A
	Age * Ever smoked	0.02 (0.02)	0.95	.343	N/A
	Age * Depression	-0.04 (0.02)	-2.28	.023	N/A
Model-based planning	Age * Low SES	0 (0.02)	0.08	.935	N/A
	Age * Less education	-0.03 (0.02)	-1.56	.118	N/A
	Age * Depression	-0.02 (0.02)	-1.31	.189	N/A
Subjective memory problems	Age * Tinnitus	-0.06 (0.04)	-1.60	.110	0.94 [0.87, 1.01]
	Age * Small soc. network	-0.07 (0.04)	-1.96	.050	0.93 [0.86, 1]
	Age * Low SES	-0.05 (0.04)	-1.28	.200	0.95 [0.88, 1.03]
	Age * Loneliness	-0.01 (0.04)	-0.37	.712	0.99 [0.92, 1.06]
	Age * Less exercise	-0.03 (0.04)	-0.88	.380	0.97 [0.89, 1.04]
	Age * Less education	-0.07 (0.04)	-1.90	.057	0.93 [0.86, 1]

Cognitive outcome	Interaction	*β (SE)*	*t/z*	*P*	OR [95% CI]
	Age * History of stroke	-0.01 (0.15)	-0.09	.932	0.99 [0.73, 1.34]
	Age * Hearing handicap	-0.1 (0.04)	-2.65	.008	0.9 [0.84, 0.97]
	**Age * Ever smoked**	**-0.14 (0.04)**	**-3.65**	**< .001**	**0.87 [0.81, 0.94]**
	Age * Depression	0.03 (0.04)	0.87	.386	1.03 [0.96, 1.12]

References

1. Donegan KR, Brown VM, Price RB, et al. Using smartphones to optimise and scale-up the assessment of model-based planning. *Commun Psychol*. 2023;1(1):1-15. doi:10.1038/s44271-023-00031-y

2. Parra MA, Abrahams S, Logie RH, Méndez LG, Lopera F, Della Sala S. Visual short-term memory binding deficits in familial Alzheimer's disease. *Brain*. 2010;133(9):2702-2713. doi:10.1093/brain/awq148

3. Bowie CR, Harvey PD. Administration and interpretation of the Trail Making Test. *Nat Protoc*. 2006;1(5):2277-2281. doi:10.1038/nprot.2006.390

4. Brockmole JR, Parra MA, Sala SD, Logie RH. Do binding deficits account for age-related decline in visual working memory? *Psychonomic Bulletin & Review*. 2008;15(3):543-547. doi:10.3758/PBR.15.3.543

5. Killin L, Abrahams S, Parra MA, Della Sala S. The effect of age on the FCSRT-IR and temporary visual memory binding. *Int Psychogeriatr*. 2018;30(3):331-340. doi:10.1017/S104161021700165X

6. Parra MA, Della Sala S, Logie RH, Morcom AM. Neural correlates of shape–color binding in visual working memory. *Neuropsychologia*. 2014;52:27-36. doi:10.1016/j.neuropsychologia.2013.09.036

7. Psychology Software Tools Inc. E-Prime. Published online 2012.

8. Tombaugh TN. Trail Making Test A and B: normative data stratified by age and education. *Arch Clin Neuropsychol*. 2004;19(2):203-214. doi:10.1016/S0887-6177(03)00039-8

9. Daw ND, Gershman SJ, Seymour B, Dayan P, Dolan RJ. Model-Based Influences on Humans' Choices and Striatal Prediction Errors. *Neuron*. 2011;69(6):1204-1215. doi:10.1016/j.neuron.2011.02.027

10. Bates D, Mächler M, Bolker B, Walker S. Fitting Linear Mixed-Effects Models Using lme4. *Journal of Statistical Software*. 2015;67:1-48. doi:10.18637/jss.v067.i01
